# Supplementary material for: Genetic diversity and population structure of soybean (Glycine max (L.) Merril) germplasm
Source: PLoS One. 2025 May 8;20(5):e0312079. doi: 10.1371/journal.pone.0312079 (PMC12061401; doi:10.1371/journal.pone.0312079)
Supplement: S1 Table — (DOCX) [file pone.0312079.s001.docx]

**S1Table**. Soybean accessions used in the study with their respective origins

| Sn | Accession | Code | Source | Origin |
| --- | --- | --- | --- | --- |
| 1 | TGx_1951-4F | SY001 | 22AVT01 | IITA |
| 2 | TGx_1993-18FN | SY002 | 22AVT01 | IITA |
| 3 | TGx_2015-2E | SY003 | 22AVT01 | IITA |
| 4 | TGx2108-02GN | SY004 | 22AVT01 | IITA |
| 5 | TGx2120-111GN | SY005 | 22AVT01 | IITA |
| 6 | TGx2102-01FN | SY006 | 22AVT01 | IITA |
| 7 | TGx1987-9FxTGx1835-10E-1-2-2-1-1 | SY007 | 22AVT01 | IITA |
| 8 | TGx1987-11FxH7-3-1-1-1-2-3-I | SY008 | 22AVT01 | IITA |
| 9 | TGx1987-11FxH7-3-1-1-1-2-6-I | SY009 | 22AVT01 | IITA |
| 10 | TGx1989-19FxTGx1987-10F-5-3-1-2-2-I | SY010 | 22AVT01 | IITA |
| 11 | TGx2111-04FN | SY011 | 22AVT01 | IITA |
| 12 | TGx_2022-3E | SY012 | 22AVT01 | IITA |
| 13 | TGx2106-2FN | SY013 | 22AVT01 | IITA |
| 14 | TGx2113-01FN | SY014 | 22AVT01 | IITA |
| 15 | TGx 2029-21F | SY015 | 22AVT01 | USDA |
| 16 | Panaroma-3 | SY016 | 22AVT01 | USDA |
| 17 | TGx 2029-7F | SY017 | 22AVT01 | USDA |
| 18 | Panaroma-1 | SY018 | 22AVT01 | USDA |
| 19 | TGx2029-31F | SY019 | 22AVT01 | USDA |
| 20 | TGx2029-49F | SY020 | 22AVT01 | USDA |
| 21 | PANORAMA-27D | SY021 | 22AVT01 | USDA |
| 22 | TGx 2029-53F | SY022 | 22AVT01 | USDA |
| 23 | TGx 2014-16FM | SY023 | 22AVT01 | IITA |
| 24 | SC-SIGNA | SY024 | 22AVT01 | SeecCo |
| 25 | SONGDA | SY025 | 22AVT01 | Ghana |
| 26 | TGx 2029-39F | SY026 | 22AVT01 | USDA |
| 27 | TGX1951-3F | SY027 | 22AVT01 | IITA |
| 28 | (TGx1987-9F/TGx1740-2F)-#F5-1025-1 | SY028 | 22AVT01 | IITA |
| 29 | (TGx1740-2F/MW1)-#F6-2002-10-15 | SY029 | 22AVT01 | IITA |
| 30 | TGx 2002 -89GN | SY030 | 22AVT01 | IITA |
| 31 | TGx 2002 -90GN | SY031 | 22AVT01 | IITA |
| 32 | TGX 2014 -64GN | SY032 | 22AVT01 | IITA |
| 33 | TGx2014-111GN | SY033 | 22AVT01 | IITA |
| 34 | TGx 2029-22F | SY034 | 22AVT01 | USDA |
| 35 | TGx 1448-2E | SY035 | 22AVT01 | IITA |
| 36 | TGx 1989-19F | SY036 | 22AVT01 | IITA |
| 37 | (TGx1740-2F/MW1)-#F6-2002-10-11 | SY037 | 22AVT01 | IITA |
| 38 | TGx 2029-20F | SY038 | 22AVT01 | USDA |
| 39 | TGx2029-27F | SY039 | 22AVT01 | USDA |
| 40 | TGX 2016 -8GN | SY040 | 22AVT01 | IITA |
| 41 | (TGx1987-10F/TGx1740-2F)-#F5-1011-8 | SY041 | 22AVT01 | IITA |
| 42 | (TGx1987-62F/MW1)-#F5-1006-15 | SY042 | 22AVT01 | IITA |
| 43 | (TGx1987-62F/MW1)-#F5-1006-22 | SY043 | 22AVT01 | IITA |
| 44 | Maksoy-4N | SY044 | 22AVT01 | Uganda |
| 45 | (TGx1740-2F/MW1)-#F6-2002-10-21 | SY045 | 22AVT01 | IITA |
| 46 | TGx1987-10FxTGx1989-19F-10-1 | SY046 | 22AVT02 | IITA |
| 47 | TGx1988-5FxTGx1989-19F-17 | SY047 | 22AVT02 | IITA |
| 48 | TGx1485-1DxTGx1989-19F-4 | SY048 | 22AVT02 | IITA |
| 49 | TGx1987-10FxTGx1989-19F-10-2 | SY049 | 22AVT02 | IITA |
| 50 | TGx1448-2ExTGx1989-19F-3 | SY050 | 22AVT02 | IITA |
| 51 | TGx1987-10FxTGx1989-19F-17 | SY051 | 22AVT02 | IITA |
| 52 | TGx1987-10FxTGx1989-19F-22 | SY052 | 22AVT02 | IITA |
| 53 | TGx1987-10FxTGx1989-19F-9 | SY053 | 22AVT02 | IITA |
| 54 | TGx1987-11FxH7-3-1-1-1-2-7-I | SY054 | 22AVT02 | IITA |
| 55 | TGx1988-5FxTGx1989-19F—16 | SY055 | 22AVT02 | IITA |
| 56 | TGx1988-5FxTGx1989-19F-18 | SY056 | 22AVT02 | IITA |
| 57 | TGx1987-10FxTGx1989-19F-13 | SY057 | 22AVT02 | IITA |
| 58 | TGx1485-1DxTGx1835-10E-2 | SY058 | 22AVT02 | IITA |
| 59 | TGx1988-5FxTGx1989-19F-20 | SY059 | 22AVT02 | IITA |
| 60 | TGx1448-2ExTGx1989-19F-1 | SY060 | 22AVT02 | IITA |
| 61 | TGx1988-5FxTGx1989-19F-13 | SY061 | 22AVT02 | IITA |
| 62 | TGx1448-2ExTGx1988-5F-1 | SY062 | 22AVT02 | IITA |
| 63 | TGx1988-5FxTGx1989-19F-9 | SY063 | 22AVT02 | IITA |
| 64 | TGx1987-10FxTGx1989-19F-18 | SY064 | 22AVT02 | IITA |
| 65 | TGx1989-45FxTGx1835-10E-3-2-2-4-3-E | SY065 | 22AVT02 | IITA |
| 66 | TGx1987-62FxTGx1988-5F-2 | SY066 | 22AVT02 | IITA |
| 67 | TGx1485-1DxTGx1835-10E-1 | SY067 | 22AVT02 | IITA |
| 68 | TGx1989-19FxPI230970-5 | SY068 | 22AVT02 | IITA |
| 69 | TGx2029-8F | SY069 | 22AVT02 | USDA |
| 70 | TGx2029-30F | SY070 | 22AVT02 | USDA |
| 71 | TGx2029-42F | SY071 | 22AVT02 | USDA |
| 72 | TGx_2024-3E | SY072 | 20AVTE | USDA |
| 73 | TGx_1987-62F | SY073 | 20AVTE | IITA |
| 74 | TGx_2024-7E | SY074 | 20AVTE | IITA |
| 75 | TGx2033-69FZ | SY075 | 22AVT02 | IITA |
| 76 | TGX1904-6F | SY076 | 22AVT02 | IITA |
| 77 | TGx_2022-1E | SY077 | 20AVTE | IITA |
| 78 | TGx_2020-4E | SY078 | 20AVTE | IITA |
| 79 | TGx_1988-5F | SY079 | 20AVTE | IITA |
| 80 | TGx_2019-2E | SY080 | 20AVTE | IITA |
| 81 | TGx_2025-15E | SY081 | 20AVTE | IITA |
| 82 | TGx_1987-10F | SY082 | 20AVTE | IITA |
| 83 | TGx_2018-1E | SY083 | 20AVTE | IITA |
| 84 | TGx_2018-2E | SY084 | 20AVTE | IITA |
| 85 | TGx_2025-7E | SY085 | 20AVTE | IITA |
| 86 | TGx_2018-3E | SY086 | 20AVTE | IITA |
| 87 | TGx_2022-2E | SY087 | 20AVTE | IITA |
| 88 | TGx_1835-10E | SY088 | 20AVTE | IITA |
| 89 | TGX1448-2E × TGX1989-19F-2 | SY089 | 22PVT01 | IITA |
| 90 | TGX1485-1D x TGX1987-10F | SY091 | 22PVT01 | IITA |
| 91 | TGX1987-10F × TGX1989-19F-5 | SY092 | 22PVT01 | IITA |
| 92 | TGX1987-10F × TGX1989-19F-7 | SY093 | 22PVT01 | IITA |
| 93 | TGX1448-2E × TGX1989-19F | SY095 | 22PVT01 | IITA |
| 94 | TGX1987-10F × TGX1989-19F | SY096 | 22PVT01 | IITA |
| 95 | TGX1987-10F × TGX1989-19F | SY097 | 22PVT01 | IITA |
| 96 | TGX1987-10F × TGX1989-19F | SY098 | 22PVT01 | IITA |
| 97 | TGX1987-10F × TGX1989-19F | SY099 | 22PVT01 | IITA |
| 98 | TGX1987-62F × TGX1989-19F | SY100 | 22PVT01 | IITA |
| 99 | TGX1987-10F × TGX1989-19F | SY101 | 22PVT01 | IITA |
| 100 | TGX1448-2E × TGX1989-19F-2 | SY102 | 22PVT01 | IITA |
| 101 | TGx 2029-4F x TGx 2029-7F | SY103 | 22PVT01 | IITA |
| 102 | TGx 2016-5E x ST SUPREMA/SOYICA P3/CIMARONA | SY104 | 22PVT01 | IITA |
| 103 | (TGx 1989-64F x ZIG x 1004) x TGx 1989-64F-6 | SY105 | 22PVT01 | IITA |
| 104 | (LG12-1902 x TGx 1740-2F) x TGx 1740-2F-3 | SY106 | 22PVT01 | IITA |
| 105 | (LG12-1902 x TGx 1740-2F) x TGx 1740-2F-5 | SY107 | 22PVT01 | IITA |
| 106 | (LG12-1902 x TGx 1740-2F) x TGx 1740-2F-16 | SY108 | 22PVT01 | IITA |
| 107 | (LG12-1902 x TGx 1740-2F) x TGx 1740-2F-19 | SY109 | 22PVT01 | IITA |
| 108 | (AS-G-003P x TGx 1740-2F) x TGx 1740-2F-2 | SY110 | 22PVT01 | IITA |
| 109 | (TGx 2001-9DM x ZIG x 1005) x TGx 2001-9DM-4 | SY111 | 22PVT01 | IITA |
| 110 | CIMARRONA OBV-(5SEC)-6-7-M(2)2001-57 x TGx 1989-19F | SY112 | 22PVT01 | IITA |
| 111 | TGX1835-10E × TGX1989-19F | SY113 | 22PVT01 | IITA |
| 112 | CIMARRONA OBV-(5SEC)-6-7-M(2)2001-57 x TGx 1989-19F-2 | SY114 | 22PVT01 | IITA |
| 113 | (TGx 1989-64F x ZIG x 1004) x TGx 1989-64F-6 | SY115 | 22PVT01 | IITA |
| 114 | (AS-G-001P x TGx 1740-2F) x TGx 1740-2F-4-2 | SY116 | 22PVT01 | IITA |
| 115 | AVT2-TGx 2002-3DM x SPSOY-2007A-068-2 | SY117 | 22PVT02 | IITA |
| 116 | AVT2-TGx 2002-3DM x SPSOY-2007A-068-3 | SY118 | 22PVT02 | IITA |
| 117 | (TGx 1740-2F x ZIG x 1006) x TGx 1740-2F-3 | SY119 | 22PVT02 | IITA |
| 118 | (TGx 1740-2F x ZIG x 1006) x TGx 1740-2F-5 | SY120 | 22PVT02 | IITA |
| 119 | (LG13-4038 x TGx 1740-2F) x TGx 1740-2F-10 | SY121 | 22PVT02 | IITA |
| 120 | TGx 2016-6E x SPSOY-2008A-00187 | SY122 | 22PVT02 | IITA |
| 121 | (AS-G-001P x TGx 1740-2F) x TGx 1740-2F-2 | SY123 | 22PVT02 | IITA |
| 122 | (AS-G-001P x TGx 1740-2F) x TGx 1740-2F-6 | SY124 | 22PVT02 | IITA |
| 123 | (AS-G-001P x TGx 1740-2F) x TGx 1740-2F-7 | SY125 | 22PVT02 | IITA |
| 124 | (AS-G-001P x TGx 1740-2F) x TGx 1740-2F-9 | SY126 | 22PVT02 | IITA |
| 125 | AVT2-TGx 2002-3DM x SPSOY-2008A-00213 | SY127 | 22PVT02 | IITA |
| 126 | (LG12-1902 x TGx 1740-2F) x TGx 1740-2F-14 | SY128 | 22PVT02 | IITA |
| 127 | (AS-G-003P x TGx 1740-2F) x TGx 1740-2F-5 | SY129 | 22PVT02 | IITA |
| 128 | (TGx 1740-2F x ZIG x 1006) x TGx 1740-2F-2 | SY130 | 22PVT02 | IITA |
| 129 | (TGx 1740-2F x ZIG x 1006) x TGx 1740-2F-4 | SY131 | 22PVT02 | IITA |
| 130 | (TGx 1740-2F x ZIG x 1006) x TGx 1740-2F-17 | SY132 | 22PVT02 | IITA |
| 131 | ST SUPREMA/SOYICA P31/ CIMARRONA x PI567090 | SY133 | 22PVT02 | IITA |
| 132 | AVT2-TGX 2001-1DM X SPSOY-2007A-068 | SY134 | 22PVT02 | IITA |
| 133 | SPSOY-2007A-030 X TGX 1951-3F | SY135 | 22PVT02 | IITA |
| 134 | TGX1448-2E × TGX1989-19F | SY136 | 22PVT02 | IITA |
| 135 | TGx 2018-1E x SPSOY-2007A-068-3 | SY137 | 22PVT02 | IITA |
| 136 | TGx 2018-1E x SPSOY-2007A-068-3 | SY138 | 22PVT02 | IITA |
| 137 | TGx 2018-3E x CIMARRONA OBV-6-7-M(2)2001-57-1 | SY139 | 22PVT02 | IITA |
| 138 | (TGx 1740-2F x ZIG x 1006) x TGx 1740-2F-8 | SY140 | 22PVT02 | IITA |
| 139 | (TGx 1740-2F x ZIG x 1006) x TGx 1740-2F-12 | SY141 | 22PVT02 | IITA |
| 140 | (LG12-4095 x TGx 1740-2F) x TGx 1740-2F-1 | SY142 | 22PVT02 | IITA |
| 141 | SPSOY-2008A-00213 x TGx 1989-19F-1 | SY143 | 22PVT02 | IITA |
| 142 | SPSOY-2008A-00187 x TGx 1989-19F-1 | SY144 | 22PVT02 | IITA |
| 143 | ST SUPREMA/SOYICA P31/ CIMARRONA x TGx 1951-3F-6-1 | SY145 | 22PVT02 | IITA |
| 144 | AVT-TGx 2001-24FM x TGx 1951-3F-1 | SY146 | 22PVT02 | IITA |
| 145 | TGx2029-5F | SY373 | 22AVT02 | USDA |
| 146 | TGx2014-22FZ | SY374 | 22AVT02 | IITA |
| 147 | TGx2033-17FZ | SY375 | 22AVT02 | IITA |

Sn: Serial number
