# Supplementary figures and images for: Genetic diversity and population structure of soybean (Glycine max (L.) Merril) germplasm
Source: PLoS One. 2025 May 8;20(5):e0312079. doi: 10.1371/journal.pone.0312079 (PMC12061401; doi:10.1371/journal.pone.0312079)

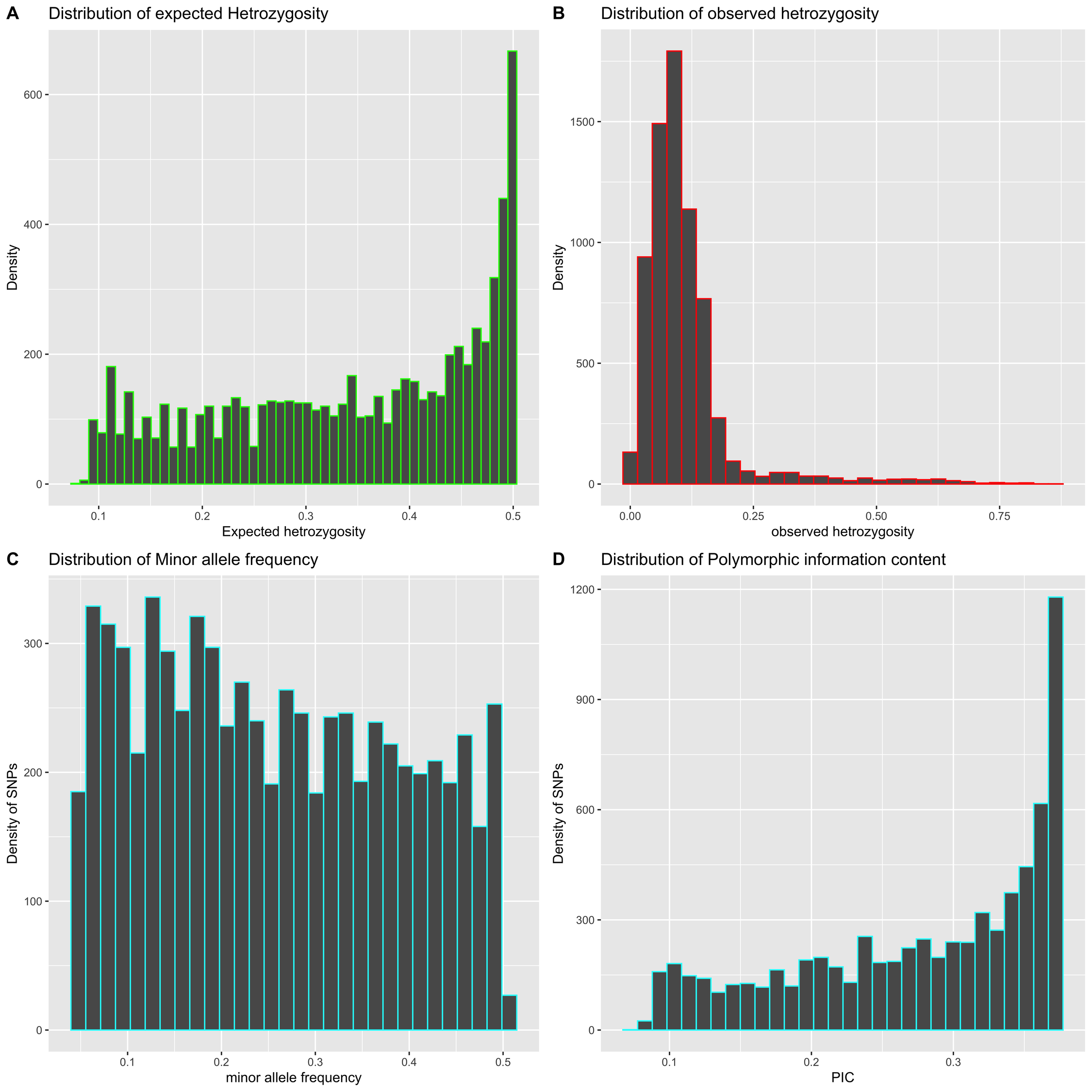

Supplement: S1 Fig — (a) Expected heterozygosity, (b) observed heterozygosity, (c) minor allele frequency and (d) polymorphic information content. (TIF) [file pone.0312079.s002.tif]

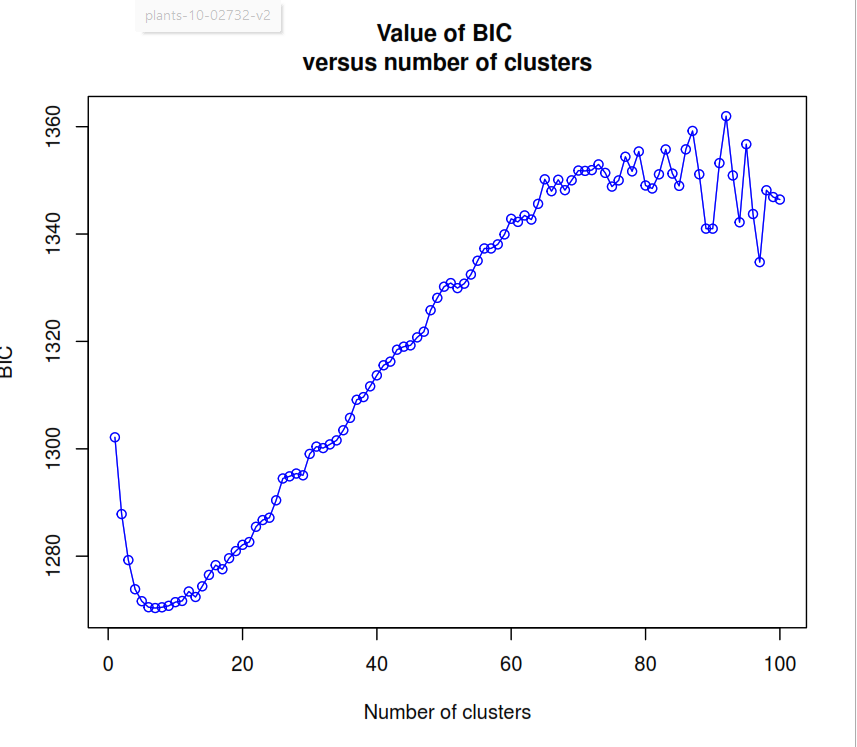

Supplement: S2 Fig — (TIF) [file pone.0312079.s003.tif]

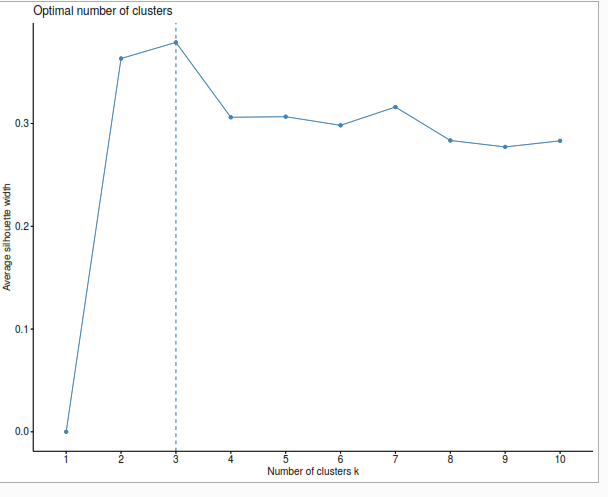

Supplement: S3 Fig — (TIF) [file pone.0312079.s004.tif]
