# Supplementary material for: Genetic diversity and population structure of soybean (Glycine max (L.) Merril) germplasm
Source: PLoS One. 2025 May 8;20(5):e0312079. doi: 10.1371/journal.pone.0312079 (PMC12061401; doi:10.1371/journal.pone.0312079)
Supplement: Attachment — Submitted filename: PONE-D-24-43093_R2_EAA.pdf [file pone.0312079.s007.pdf]

PLOS ONE

Genetic diversity and population structure of soybean (Glycine max (L.) Merril) germplasm.

--Manuscript Draft--

**Manuscript Number:**	PONE-D-24-43093R2
**Article Type:**	Research Article
**Full Title:**	Genetic diversity and population structure of soybean (Glycine max (L.) Merril) germplasm.
**Short Title:**	Soybean's genetic diversity and population structure
**Corresponding Author:**	TENENA SILUE, Doctorate<br>International Institute of Tropical Agriculture<br>IBADAN, NIGERIA
**Keywords:**	Genetic diversity; DArTseq; SNPs; soybean; DAPC; admixture
**Abstract:**	Soybean (Glycine max (L.) Merril) is a significant legume crop for oil and protein. However, its yield in Africa is less than half the global average resulting in low production, which is inadequate for satisfying the continent's needs. To address this disparity in productivity, it is crucial to develop new high-yielding cultivars by utilizing the genetic diversity of existing germplasms. Consequently, the genetic diversity and population structure of various soybean accessions were evaluated in this study. In pursuit of this objective, a collection of 147 soybean accessions was genotyped via the Diversity Array Technology Sequencing method. This method enables high-throughput analysis of single-nucleotide polymorphisms (SNPs), resulting in the identification of 7,083 high-quality SNPs distributed throughout the soybean genome. The average values observed for polymorphism information content (PIC), minor allele frequency, expected heterozygosity and observed heterozygosity were 0.277, 0.254, 0.344, and 0.110, respectively. The soybean genotypes were categorized into four groups on the basis of model-based population structure, principal component analysis, and discriminant analysis of the principal component. Alternatively, hierarchical clustering was used to organize the accessions into three distinct clusters. Analysis of molecular variance indicated that the genetic variance within the populations exceeded the variance among them. The insights gained from this study will assist breeders in selecting parental lines for genetic recombination. Overall, this study provides valuable information regarding soybean genetic diversity and lays the groundwork for conservation and genetic enhancement initiatives.
**Order of Authors:**	TENENA SILUE, Doctorate
	Paterne Angelot Agre, Ph.D
	Bunmi Olasanmi, Ph.D
	Adeyinka Saburi Adewumi, Ph.D
	Idris Ishola Adejumobi, Ph.D
	Abush Tesfaye Abebe, Ph.D
**Response to Reviewers:**	Authors’ Responses to Reviewers' comments<br><br>Reviewer #1: Dear …<br>The researchers analyzed the genetic diversity in soybean accessions using DArt technology. There isn't much new information available on the subject. The following adjustments are needed:<br><br>Abstract<br>1.The aim and the methods should be joined into a single phrase<br>Response: Thanks for your valuable observation. The aim and the methods have been joined into a single phrase. (Reference: Lines 22-25).<br>2.It would be helpful to include a brief conclusion about the obtained results<br>Response: This has adequately taken care off (Lines 37-39).

Powered by Editorial Manager® and ProduXion Manager® from Aries Systems Corporation

Introduction

1.This section is too long and should be summarized

Response: Thank you for your observation. This section has been reduced to provide the most relevant information.

2.It is suggested that you use the most current references that you can find.

Response: Thank you for this comment, we have included as many recent references that are relevant to our work as possible while retaining the old references that are highly important in the revised version of the manuscript.

3. The authors should describe the DNA molecular marker related to this study

Response: Thank you for your observation, the requested information has been provided. (Lines 117-127) in the revised manuscript.

4.Detailed information regarding methodologies for genetic diversity should be provided.

Response: Thank you for your observation. The methodologies for assessing genetic diversity in crops was documented in line 68 – 83.

5.The authors should provide the gap and hypothesis of this study.

Response: Thank you for your comment. The requested information has been updated. (Lines 136-142).

6.The authors should expressly indicate in a single sentence the originality of the work

Response: Thank you for the comments. This aspect has been addressed. (Lines 142-144).

7.What distinguishes this study is that the authors identified new tasks or linkages related to their investigation.

Response: We appreciate your comments. We have included the statement showing how our work was unique following the identified gap in the IITA soybean breeding program.

8.Both general and specific goals should be documented

Response: Thank you for your comment. This observation has been provided (Lines 168-169).

Material and methods

9.The tissue type used for DNA extraction should be described

Response: Thank you for your comment. The description of the tissue type used for DNA extraction was documented in lines 182-184.

10.The name of the commercial kit should be included

Response: Thank you for your comment. We have included the names of the commercial kits as requested in the revised manuscript.

11.It is essential to cite your sources or references for each approach

Response: Thank you for your observation. Appropriate citations following the format of the PLOSONE journal have been made for the sources and approaches we explored and documented in the revised version of the manuscript.

12.The manufacture of all instruments and materials should be stated

Response: Thank you for your observation. We have tried our best to include the manufacturer of the instruments and materials that we could find as requested in the revised version of the manuscript. Our limitation was because the genotyping procedure (DNA extraction and genomic scan) were all performed by the DArT laboratory.

13.Why did not the authors perform all measurements of genetic diversity indices like Na, Ne, and I?

Response: we appreciate your contribution in ensuring our manuscript is better improved. Our understanding of Na, Ne, and I points to population size, effective sample size, and inbreeding depression, respectively which we do not believe may provide newer and different information than the diversity parameters (allele frequency, PIC, HO, He, 1/D, H', and A) we have explored in the study.

14.The authors should perform the computation of genetic diversity index for population

Response: Thank you so much for this suggestion. We have included more population diversity assessment indices including the Shannon-Wiever index, inverse Simpson index, and Alpha diversity index to verify the results obtained from the marker diversity indices.

Results and discussion

Powered by Editorial Manager® and ProduXion Manager® from Aries Systems Corporation

	15.This section is simply written and should be detailed<br>Response: Thanks so much for your observation. We have included more interpretations and tailored our discussion to the interpreted results in the revised version of the manuscript.<br>16.The results of tables should be detailed<br>Response: Thanks so much for your suggestion. The results in the tables have been improved to include a better caption and footnote adjustment in the revised version of the manuscript as requested.<br>17.All abbreviation should be written in full name<br>Response: Thank you for your comment. Abbreviations in the manuscript have been fully written as requested in the revised manuscript.<br>18.When describing the maximum and minimum values of the attributes, the authors should use scored data.<br>Response: Thank you for your comment. We have included minimum, mean, and maximum values in Table 1. The information in this table was drawn from quantitative data.<br>19.Every caption should be enhanced.<br>Response: Thank you for your observation. We have enhanced the captions in the Tables and figures within the revised manuscript as requested.<br>20.The Delta K figure of genetic structure should be spotted<br>Response<br>We appreciate your comment. We have incorporated the information of obtained in the manuscript and added the optimal cluster information from the silhouette plot for our HC as a supplementary file.<br><br>21.The discussion did not have enough conviction. The analysis is a significant part of the remarks; yet, the majority of them merely repeat the findings without critically analyzing the facts. Please explain to readers how the authors' findings relate to those of other studies. Detailed information must be supplied on how each recorded parameter effects high or poor results. Their description should include all of the ramifications of setting high or low values for each of these parameters. The authors should interpret all measurements like PIC, He, and multivariate analysis.<br>Response: Thank you so much for your observation. We have revised the discussion section of the manuscript to ensure it provides adequate conviction to the readers as requested in the revised manuscript.<br><br>Conclusion<br>22.This section's authors should present a concise summary of the most important findings in an easy-to-read format. This area should be explored more in future studies<br>Response: Thank you for your observation. We have provided the summary of the most important findings in a concise manner as requested.
**Additional Information:**	
**Question**	**Response**
**Financial Disclosure**<br><br>Enter a financial disclosure statement that describes the sources of funding for the work included in this submission. Review the submission guidelines for detailed requirements. View published research articles from *PLOS ONE* for specific examples.<br><br>This statement is required for submission and **will appear in the published article** if the submission is accepted. Please make sure it is accurate.	Yes

**Funded studies**<br>Enter a statement with the following details:<br>• Initials of the authors who received each award<br>• Grant numbers awarded to each author<br>• The full name of each funder<br>• URL of each funder website<br>• Did the sponsors or funders play any role in the study design, data collection and analysis, decision to publish, or preparation of the manuscript?<br><br>Did you receive funding for this work?	
Please add funding details.<br>as follow-up to "**Financial Disclosure**<br><br>Enter a financial disclosure statement that describes the sources of funding for the work included in this submission. Review the submission guidelines for detailed requirements. View published research articles from *PLOS ONE* for specific examples.<br><br>This statement is required for submission and **will appear in the published article** if the submission is accepted. Please make sure it is accurate.<br><br>**Funded studies**<br>Enter a statement with the following details:<br>• Initials of the authors who received each award<br>• Grant numbers awarded to each author<br>• The full name of each funder<br>• URL of each funder website<br>• Did the sponsors or funders play any role in the study design, data collection and analysis, decision to publish, or preparation of the manuscript?<br><br>Did you receive funding for this work?"	The field experiment was funded by 'IITA/USAID Genetic Improvement in Soy project, PJ-2315.The funders of this manuscript (IITA/USAID Genetic Improvement in Soy project, PJ-2315) had no role in the study design, data collection and analysis, decision to publish, or preparation of the manuscript.
Please select the country of your main research funder (please select carefully as in some cases this is used in fee calculation).	NIGERIA - NG

as follow-up to "**Financial Disclosure**<br><br>Enter a financial disclosure statement that describes the sources of funding for the work included in this submission. Review the submission guidelines for detailed requirements. View published research articles from *PLOS ONE* for specific examples.<br><br>This statement is required for submission and **will appear in the published article** if the submission is accepted. Please make sure it is accurate.<br><br>**Funded studies**<br>Enter a statement with the following details:<br>• Initials of the authors who received each award<br>• Grant numbers awarded to each author<br>• The full name of each funder<br>• URL of each funder website<br>• Did the sponsors or funders play any role in the study design, data collection and analysis, decision to publish, or preparation of the manuscript?<br><br>Did you receive funding for this work?"	
**Competing Interests**<br><br>Use the instructions below to enter a competing interest statement for this submission. On behalf of all authors, disclose any competing interests that could be perceived to bias this work—acknowledging all financial support and any other relevant financial or non-financial competing interests.<br><br>This statement is required for submission and **will appear in the published article** if the submission is accepted. Please make sure it is accurate and that any funding sources listed in your Funding Information later in the submission form are also declared in your Financial Disclosure statement.	The authors have declared that no competing interests exist.

View published research articles from [*PLOS ONE*] for specific examples.<br><br>**NO authors have competing interests**<br><br>Enter: *The authors have declared that no competing interests exist.*<br><br>**Authors with competing interests**<br><br>Enter competing interest details beginning with this statement:<br><br>*I have read the journal's policy and the authors of this manuscript have the following competing interests: [insert competing interests here]*<br><br>* typeset	
**Ethics Statement**<br><br>Enter an ethics statement for this submission. This statement is required if the study involved:<br><br>• Human participants<br>• Human specimens or tissue<br>• Vertebrate animals or cephalopods<br>• Vertebrate embryos or tissues<br>• Field research<br><br>Write "N/A" if the submission does not require an ethics statement.<br><br>General guidance is provided below. Consult the submission guidelines for detailed instructions. **Make sure that all information entered here is included in the Methods section of the manuscript.**	N/A

**Format for specific study types**<br><br>**Human Subject Research (involving human participants and/or tissue)**<br>• Give the name of the institutional review board or ethics committee that approved the study<br>• Include the approval number and/or a statement indicating approval of this research<br>• Indicate the form of consent obtained (written/oral) or the reason that consent was not obtained (e.g. the data were analyzed anonymously)<br><br>**Animal Research (involving vertebrate animals, embryos or tissues)**<br>• Provide the name of the Institutional Animal Care and Use Committee (IACUC) or other relevant ethics board that reviewed the study protocol, and indicate whether they approved this research or granted a formal waiver of ethical approval<br>• Include an approval number if one was obtained<br>• If the study involved *non-human primates*, add *additional details* about animal welfare and steps taken to ameliorate suffering<br>• If anesthesia, euthanasia, or any kind of animal sacrifice is part of the study, include briefly which substances and/or methods were applied<br><br>**Field Research**<br><br>Include the following details if this study involves the collection of plant, animal, or other materials from a natural setting:<br>• Field permit number<br>• Name of the institution or relevant body that granted permission	
**Data Availability**<br><br>Authors are required to make all data underlying the findings described fully available, without restriction, and from the time of publication. PLOS allows rare exceptions to address legal and ethical concerns. See the PLOS Data Policy and FAQ for detailed information.	Yes - all data are fully available without restriction

A Data Availability Statement describing where the data can be found is required at submission. Your answers to this question constitute the Data Availability Statement and **will be published in the article**, if accepted.<br><br>**Important:** Stating 'data available on request from the author' is not sufficient. If your data are only available upon request, select 'No' for the first question and explain your exceptional situation in the text box.<br><br>Do the authors confirm that all data underlying the findings described in their manuscript are fully available without restriction?	
**Describe where the data may be found in full sentences. If you are copying our sample text, replace any instances of XXX with the appropriate details.**<br><br>• If the data are **held or will be held in a public repository**, include URLs, accession numbers or DOIs. If this information will only be available after acceptance, indicate this by ticking the box below. For example: *All XXX files are available from the XXX database (accession number(s) XXX, XXX.*).<br>• If the data are all contained **within the manuscript and/or Supporting Information files**, enter the following: *All relevant data are within the manuscript and its Supporting Information files.*<br>• If neither of these applies but you are able to provide **details of access elsewhere**, with or without limitations, please do so. For example:<br><br>*Data cannot be shared publicly because of [XXX]. Data are available from the XXX Institutional Data Access / Ethics Committee (contact via XXX) for researchers who meet the criteria for access to confidential data.*<br><br>*The data underlying the results presented in the study are available from (include the name of the third party*	All relevant data are within the manuscript and its Supporting Information files

and contact information or URL).<br>• This text is appropriate if the data are owned by a third party and authors do not have permission to share the data.<br><br>* typeset	
Additional data availability information:	Tick here if the URLs/accession numbers/DOIs will be available only after acceptance of the manuscript for publication so that we can ensure their inclusion before publication.

Powered by Editorial Manager® and ProduXion Manager® from Aries Systems Corporation

Manuscript

Click here to access/download;Manuscript;Revised manuscript - Clean copy.docx

RESEARCH ARTICLE

Genetic diversity and population structure of soybean (*Glycine max* (L.) Merril) germplasm

Tenena Silue[1,2,*], Paterne Angelot Agre[2], Bunmi Olasanmi[3], Adeyinka Saburi Adewumi[2], Idris Ishola Adejumobi[2] and Abush Tesfaye Abebe [2,*]

[1] Pan African University Life and Earth Sciences Institute (including Health and Agriculture), University of Ibadan, Ibadan, Oyo State, Nigeria

[2] International Institute of Tropical Agriculture (IITA), Ibadan, Oyo State, Nigeria

[3] Department of Crop and Horticultural Sciences, University of Ibadan, Ibadan, Oyo State, Nigeria

* Corresponding authors

Email: at.abebe@cgiar.org, siluetenenan@yahoo.fr

Abstract

Soybean (*Glycine max* (L.) Merril) is a significant legume crop for oil and protein. However, its yield in Africa is less than half the global average resulting in low production, which is inadequate for satisfying the continent's needs. To address this disparity in productivity, it is crucial to develop new high-yielding cultivars by utilizing the genetic diversity of existing germplasms. Consequently, the genetic diversity and population structure of various soybean accessions were evaluated in this study. To achieve this objective, a collection of 147 soybean accessions was genotyped using the Diversity Array Technology Sequencing method, enabling high-throughput analysis of 7,083 high-quality single-nucleotide polymorphisms (SNPs) distributed across the soybean genome. The average values observed

for polymorphism information content (PIC), minor allele frequency, expected heterozygosity and observed heterozygosity were 0.277, 0.254, 0.344, and 0.110, respectively. The soybean genotypes were categorized into four groups on the basis of model-based population structure, principal component analysis, and discriminant analysis of the principal component. Alternatively, hierarchical clustering was used to organize the accessions into three distinct clusters. Analysis of molecular variance indicated that the genetic variance (77%) within the populations exceeded the variance (23%) among them. The insights gained from this study will assist breeders in selecting parental lines for genetic recombination. The present study demonstrates that soybean improvement is viable within the IITA breeding program, and its outcome will help to optimize the genetic enhancement of soybeans.

Keywords: DArTSeq, SNPs, DAPC, admixture, AMOVA

Introduction

Soybean (*Glycine max* (L.) Merril) is a self-pollinated crop from the Fabaceae family with a diploid chromosome number of 2n = 40 [1]. It is one of the world's major legumes and oil crops in terms of production and trade [2]. Soybean contains approximately 38–42% high-quality protein and 18–20% oil rich in essential fatty acids [3]. In Nigeria, soybean is used to produce nutritious drinks known as “soymilk” and “awara” (soybean cake). It is also a crucial ingredient in poultry and fish feed and is also used in infant meals. The oil is utilized in cooking and as a base for mayonnaise, margarine, salad dressings, and shortening [4].

Globally, soybean cultivation covers approximately 121 million hectares, with an estimated total production of 334 million tons annually [5]. The top three producers (Brazil, the United States, and Argentina) together contribute 73% of the world’s production. In Africa, approximately 2.55 million hectares are dedicated to soybean cultivation, with an average productivity of 1,348 kg per hectare. South Africa, Nigeria, and Zambia are the leading

producers on the continent, with annual production rates of 1.32, 0.73, and 0.35 million tons, respectively [6].

Soybean cultivation in Africa typically yields less than 1.5 t. ha^{-1}, which is significantly below the potential yield of over 3 t. ha^{-1} [7]. This low productivity might be attributed to various factors, including the limited availability of high-yielding and climatically resilient improved varieties, poor soil fertility, diseases and pests, high pod shattering, inadequate agronomic practices, and particularly drought caused by inconsistent rainfall [8]. Therefore, there is a need for improved soybean varieties that are resilient to these biotic and abiotic stresses [9]. Furthermore, genetic diversity in many crops has decreased over time as commercial plant breeding focuses on enhancing one or a few traits and/or uses a limited number of exceptional genotypes to create a breeding population [10].

Exploiting and conserving crop genetic diversity is essential for developing new cultivars with desirable traits [11]. Assessing genetic diversity within germplasm is essential for expanding a core collection and enhancing germplasm utilization in breeding programs [2]. Identifying genes regulating beneficial traits in existing populations such as breeding lines, cultivars, landraces, and wild relatives, is crucial for improving current varieties and promoting sustainable farming practices [12]. Additionally, understanding genetic variability within and between plant populations aids breeders in improving breeding strategies [13].

Crop variability can be assessed at both the phenotypic and genotypic levels using statistical methods to separate genetic and environmental components [14]. While morphological markers detect diversity, they are less effective than DNA markers because of their subjectivity, limited number, and environmental sensitivity [9]. DNA markers are more effective for evaluating genetic diversity, aiding in the efficient use of germplasms for conservation and crop yield improvement. Soybean genetic diversity has been assessed using various biochemical and molecular markers [15], including isozymes [16, 17], random amplified

polymorphic DNA (RAPD) [18], random fragment length polymorphism (RFLP) [19], amplified fragment length polymorphism (AFLP) [20], simple sequence repeats (SSR) [21, 25], and single nucleotide polymorphisms (SNPs). Among these, SSR markers are effective for identifying genetic relationships within soybean populations [26, 28], polymerase chain reaction (PCR) based amplification can sometimes result in sequence artefacts, complicating genotyping [29, 34]. RAPD markers, although useful, have limitations like low discriminatory power and high genotyping costs [35]. The rise of next-generation sequencing (NGS) has made SNP markers the preferred choice for studying genetic diversity due to their precision, cost-effectiveness, and even distribution across the genome [36, 37].

According to Fischer et al. [38], SNP markers are the most effective among the molecular markers used as genomic resources for identifying variations in crop varieties, including soybeans. The high abundance in the genome and the ability to identify variation at a single locus make SNP markers outstanding among the marker groups explored for genomic activity [38]. Studies have generated extensive SNP data to explore genetic diversity between wild and cultivated soybeans, focusing on wild soybean allele diversity [39], uncovering valuable genetic information for breeding efforts [40], examining the genetic diversity and structure [41,42], and creating detailed haplotype maps using whole genome sequencing [43, 44].

Diversity Array Technology (DArT) is a high-throughput genotyping method that provides cost-effective, scalable, whole-genome profiling, making it a versatile tool for various genetic applications [45]. It offers better coverage and fewer missing data compared to other next-generation sequencing (NGS) platforms and has been successfully used in crops like soybean [9–47, 48], maize [49], wheat [50, 51], cowpea and [52, 53], sorghum [54], and garlic [55].

SNP markers, which are abundant and stable across the genome, are ideal for studying genetic variation and population structure [56]. While SNPs have been widely used to assess

genetic diversity in soybeans globally, there is limited research on African germplasm, especially IITA's breeding materials. Understanding the genetic diversity of IITA's soybean germplasm can reveal distinct sub-populations, and historical breeding patterns, and guide future breeding efforts [57, 58]. Population structure analysis is crucial for avoiding inbreeding, optimizing parent selection, and enhancing breeding outcomes. This study uniquely explores the genetic diversity of IITA's soybean germplasm using SNP markers, filling a critical gap in research on African soybean breeding populations.

Given the importance of genetic diversity assessment in optimizing breeding strategy for the IITA soybean improvement and the utility of SNP markers for improved precision for genetic diversity assessment, this study aims to assess the genetic diversity and population structure of IITA soybean germplasm using SNP markers. This will provide valuable insights for enhancing soybean breeding programs in SSA and contribute to the broader goal of improving food security in the region.

Materials and methods

Plant materials, planting, and leaf sampling and DNA extraction procedure

A total of 147 soybean accessions, comprised of 130 genotypes from the IITA soybean breeding program, and 17 genotypes sourced from the United States Department of Agriculture (USDA) genetic resource center (14 genotypes), Ghana (1 genotype), Uganda (1 genotype), and a private seed company (SeedCo) (1 genotype) (list of germplasms, S1 Table), were selected and utilized for a molecular-based diversity assessment.

The 147 soybean accessions were sown and grown to the seedling stage in a screen house at IITA station Ibadan, Nigeria at 243 m.a.s., 7°30′8″N longitude and 3°54′37″E latitude. Three weeks after planting, five-leaf discs 5 mm in diameter from young and healthy leaves

were collected via a biopsy curette from the leaf blades of each of the 147 genotypes. The leaf samples were placed into 96-well collection plates (12 x 8-strip tubes per 96-deep well plate) and lyophilized using a Labconco Freezone 6 plus dryer. The lyophilized leaf samples were sent to Diversity Array Technology (DArT)®, Canberra, Australia, for DNA extraction, library construction, and SNP marker development.

The DNA was extracted using a technique developed by Intertek-AgriTech (http://www.intertek.com/agriculture/agritech/) and based on the LGC oKtopure™ automated high-throughput 'sbeadex™' DNA extraction and purification system (https://www.biosearchtech.com/). Magnetic separation is used in the 'sbeadex™' technique to prepare nucleic acids. The first stage in this process is to homogenize leaf tissue samples in 96 deep-well plates using steel bead grinding. The ground tissue is treated with a DNA extraction buffer using LGC's 'sbeadex™' kit for plant DNA preparation (https://www.biosearchtech.com/). Finally, super-paramagnetic particles coated with 'sbeadexTM' surface chemistry that catches nucleic acids from a sample are used to purify extracted DNA. Purified DNA is eluted and used in downstream procedures.

High-throughput genotyping was conducted in 96 plex DArTseq protocol, and SNPs were called using the DArT's proprietary software, DArTSoft, as described by Killian et al. [46]. Each sequencing result's reads and tags were aligned to the soybean reference genome [59].

SNP Marker Quality Control

Single-row format data from DArT were initially converted into HapMap and variant call format (VCF) formats using KDcompute (https://kdcompute.seqart.net/kdcompute, accessed on 07/06/2024). SNP-derived markers were then first filtered using PLINK 1.9 and VCFtools, on the basis of the call rate of the raw data [60]. The SNP markers with call rates ranging from 0.80 to 1.0 were selected for further quality control analyses. Duplicate SNP markers across the chromosomes were removed. This process involved removing markers with

minor allele frequencies of less than 5%, markers and genotypes with more than 20% missing data, and those with a low coverage read depth of less than 5 [61, 62].

Statistical Analyses

The structure and pattern of genetic diversity within soybean genotypes were assessed via genotypic data generated on the basis of SNP markers. VCFtools and PLINK 1.9 were used to estimate summary statistics such as observed and expected heterozygosity, minor allele frequency (MAF), and polymorphic information content (PIC). The genotypic data was formatted in dosage format (0,1,2) using the recodeA function in Plink, where 0 is the homozygote reference, 1 is the homozygote alternative, and 2 is the heterozygote. The generated dosage format was then analyzed with the vegan library in R to estimate several genetic diversity indices, including the Shannon-Wiener index (H′), the inverse Simpson index (1/D) and the Alpha diversity index (A). These indices were used to assess the soybean genotypes' overall genetic diversity and allelic richness, following the methodology outlined by Oksanen et al. [63]. The SNP distribution and density plot of the SNP markers across the 20 chromosomes of the soybean genome was constructed via the CMplot package [64]. The SNP markers data were subjected to population structure analysis following the method described by Agre et al. [65]. By testing cluster numbers ranging from 2 to 10, the optimal number of clusters was identified through k-means analysis, employing cross-validation on the basis of the Bayesian information criterion (BIC). Each soybean genotype was assigned to its respective cluster if it had at least 70% ancestry probability. Genotypes with less than 70% ancestry were considered as admixed. The diversity pattern revealed through population structure analysis was further supported by discriminant analysis of the principal component (DAPC) via the Adegenet package in R [66]. DAPC, which uses the k-means clustering method, aims to minimize variance within clusters while maximizing variance between clusters [67]. Pairwise genetic dissimilarity distances (identity-by-state, IBS) were calculated via the

Jaccard method, implemented in the Philentropy R package [68]. A Ward's minimum variance hierarchical cluster dendrogram was then constructed from the Jaccard dissimilarity matrix using the Analyses of Phylogenetics and Evolution (APE) package in R [69]. Principal component analysis (PCA) was subsequently conducted to determine the genetic relationships among 147 soybean genotypes via the FactoMineR [70] and FactoExtra R packages [71]. Molecular variance analysis (AMOVA) and calculation of the coefficient of genetic differentiation among populations (PhiPT) were performed to investigate the distribution of genetic diversity among and within hierarchical populations via GenAlEx software (v.6.51) [72].

Results

Genetic diversity indices

A total of 59,126 SNP markers from the 147 soybean genotypes were originally generated via the Diversity Arrays Technology (DArT) platform. The transformation of these allelic sequences into genotypic data resulted in a raw data file of 53,418 SNPs, and after quality control analysis (SNP filtering), only 7,083 SNP markers were retained for further analyses. These markers were unequally distributed across the 20 soybean chromosomes (Fig 1; Table 1). The genome-wide SNP density plot indicated that chromosome 18 had the highest concentration of SNPs, accounting for 7.6% of the total number of markers with 538 SNPs. In contrast, chromosome 12 had the lowest concentration, with only 3.17% of the SNPs, totaling 225 markers (Fig 1). The diversity indices for the SNP marker presented a polymorphic information content (PIC) value of 0.277, ranging from 0.262 to 0.293. The MAF averaged 0.254 across all the markers. The observed heterozygosity (Ho) ranged from 0.093 to 0.124, with an average value of 0.110. The expected heterozygosity (He) varied between 0.322 and 0.371, with an average of 0.344 (Table 1 and S1 Fig). The Shannon diversity index (H′) index ranged from 8.505 to 8.704, with a mean value of 8.597. The inverse Simpon's index (1/D) had

an average of 5349.145, with a range from 4903.021 to 5824.754. The Alpha diversity index (A) varied between 4218.816 and 7568.063, with a mean value of 4950.72.

Fig 1. Distribution and density of filtered SNPs across 20 soybean chromosomes. The horizontal axis represents the chromosome length, the SNP density in each region is indicated at the bottom right

Table 1. Summary statistics of diversity indices of 147 soybean accessions based on SNP markers.

Chr	Nbr of SNPs	Ho	He	MAF	PIC	H′	1/D	A
1	260	0.116	0.359	0.267	0.287	5.304	198.66	188.77
2	334	0.111	0.335	0.250	0.269	5.546	253.235	235.059
3	345	0.105	0.338	0.246	0.272	5.579	262.490	242.013
4	328	0.094	0.322	0.231	0.262	5.555	256.267	231.879
5	285	0.104	0.349	0.256	0.281	5.382	215.95	198.54
6	462	0.095	0.34	0.251	0.274	5.886	356.442	325.612
7	296	0.093	0.323	0.230	0.263	5.438	228.309	205.593
8	440	0.108	0.334	0.241	0.270	5.838	339.115	313.574
9	374	0.108	0.371	0.288	0.293	5.619	271.903	256.762
10	247	0.108	0.348	0.259	0.278	5.225	184.079	170.973
11	274	0.105	0.339	0.250	0.272	5.340	206.522	189.444
12	225	0.113	0.354	0.265	0.284	5.139	168.733	157.169
13	470	0.124	0.357	0.273	0.284	5.870	349.287	332.682
14	399	0.112	0.357	0.265	0.287	5.712	299.103	332.682
15	435	0.112	0.345	0.249	0.278	5.820	333.216	310.035
16	463	0.119	0.333	0.238	0.270	5.876	352.129	326.264
17	290	0.124	0.357	0.269	0.284	5.405	220.020	207.293
18	538	0.114	0.344	0.255	0.277	6.002	399.211	37098
19	310	0.112	0.353	0.232	0.284	5.418	223.069	208.533
20	308	0.111	0.336	0.250	0.271	5.460	232.811	216.033
**Min**	225	0.094	0.322	0.230	0.262	8.505	4903.021	4218.816
**Mean**	354.15	0.110	0.344	0.254	0.277	8.597	5349.145	4950.728
**Max**	538	0.124	0.371	0.228	0.293	8.704	5824.754	7568.063

Chr: chromosome, Nbr of SNPs: Number of single nucleotide polymorphism, Ho: observed heterozygosity; He: expected heterozygosity; MAF: minor allelic frequency; PIC: polymorphism information content, H′: Shannon-Wiener index, 1/D: inverse Simpson index, A: Alpha diversity index, Max= maximum, Min= minimum

Population structure of 147 soybean breeding lines

Various complementary methods, including a model-based Bayesian approach in ADMIXTURE, DAPC, and PCA), were utilized to analyze the population structure of the 147 soybean accessions. Based on the admixture results, four subpopulations (K=4) were identified (Fig 2). Similarly, DAPC revealed four genetic groups (Fig 3), following a sharp decline in the Bayesian information criterion (BIC) versus the number of cluster plots (S2 Fig). There was a disparity in how the soybean accessions were assigned to the identified genetic groups between the ADMIXTURE and DAPC results. This disparity may be related to the DAPC analysis, which assigned the 147 soybean genotypes into distinct groups. In contrast, ADMIXTURE assigned 57% of the accessions (84 genotypes) to the four subpopulations on the basis of a membership probability of 70%, whereas the remaining 43% (63 lines) of the collection were classed as admixtures.

Fig 2. Population structure of 147 soybean breeding lines from the IITA breeding program, Ibadan on the basis of ADMIXTURE analysis with the subpopulations set at K = 4 via 7,083 high-quality SNPs. The colors correspond to the four subpopulations: Subpopulation 1 (red), Subpopulation 2 (blue), Subpopulation 3 (green) and Subpopulation 4 (cyan), determined by a membership coefficient greater than70%.

Fig 3. Summary of discriminant analysis of principal component (DAPC) for 147 soybean accessions, illustrating the ordination plot of DAPC for the four groups. Eigenvalues are displayed in the upper-left inset. Genetic groups or clusters are represented by distinct colors and inertia ellipses, with individual genotypes indicated by dots.

Hierarchical cluster (HC) analysis grouped all 147 soybean genotypes into three major genetic groups or clusters (Fig 4 and S3 Fig). Cluster 1 contained 87 genotypes, predominantly IITA breeding lines, except a single genotype, 'SONGDA' introduced from Ghana, which was originally an IITA breeding line sent to Ghana in variety trials. The 86 IITA genotypes were mainly TGx (Tropical *Glycine max*) varieties or progenies resulting from crosses between two TGx parental lines (S1 Table). The HC analysis grouped these 87 genotypes into Cluster 1, while the DAPC divided them into two distinct clusters, represented as Clusters 3 and 4 (Figure

3). According to the ADMIXTURE analysis, 36 of the 87 genotypes in Cluster 1, including the unique Ghana genotype, were classified as admixtures. The remaining 51 accessions were assigned to the blue and cyan groups, with 34 and 17 genotypes, respectively (Figure 2). Cluster 2 comprised 34 accessions, including 16 IITA-breeding lines, 16 of the 17 genotypes sourced from the USDA soybean genetic resource center, and one variety Sc-Signa from SeedCo (a private Company) and MAKSOY-4N from Makere University, Uganda. The 16 IITA breeding lines consisted of progenies derived from various parental lines, including TGx, ZIGx, SPSOY, CIMARRONA, PI567090, SOYICA and ST SUPREMA (S1 Table). Among the 34 genotypes in Cluster 2 identified by HC analysis, 15, exclusively IITA breeding lines, were clustered by ADMIXTURE analysis in subpopulation 1 (red) (Figure 2). The remaining 19 genotypes, which included one IITA breeding line, 16 from Columbia, and the unique genotypes from SeedCo and Uganda, were assigned as admixes by ADMIXTURE analysis. On the other hand, the DAPC analysis placed all Cluster 2 genotypes into Cluster 1 (Figure 3). The 26 genotypes assigned to Cluster 3 by HC analysis included 25 IITA breeding lines and one Columbia genotype (Panaroma-3). The IITA breeding lines were a mix of pure TGx parents and backcross progenies, derived from crosses between TGx lines and other parental lines, such as ST SUPREMA, CIMARONA, SOYICA, ZIGx, LG-12, and AS-G (S1 Table). The DAPC analysis classified these 26 genotypes from Cluster 3 in the HC analysis into Cluster 2 (Fig 3). Moreover, the ADMIXTURE analysis placed 18 of them into a specific group (green) (Fig 3), whereas the remaining 8, including the unique USDA genotype (Panaroma-3), were categorized as admixtures.

Fig4. Hierarchical clustering analysis based on 7,083 DArT-SNP markers, depicting the genetic relationships among 147 soybean accessions from the IITA, Ibadan breeding programme.

Principal component analysis (PCA) revealed that the first and second components (PC1 and PC2) accounted for 45.2% and 15.9% of the total molecular variation, respectively, together explaining 61.1% of the overall observed variation (Figure 5). Although all the

genotypes within each cluster were grouped, they exhibited some heterogeneity. The genotypes classified as admixtures were identified as admixed groups in the PCA (Figure 5).

Fig 5. Principal component analysis plot showing the clustering of 147 soybean breeding accessions into four clusters. Each cluster is represented by a distinct color: Cluster 1 (red), Cluster 2 (yellow), Cluster 3 (green), Cluster 4 (blue), and admixed individuals (pink).

Genetic distance and differentiation of soybean accessions

A pairwise dissimilarity genetic distance matrix revealed that the genetic distance among the 147 soybean genotypes ranged from 0.012 to 0.452, with an average distance of 0.333. The greatest genetic distance of 0.452 was found between the USDA genotype TGx 2029-39F (Cluster 2) and two IITA breeding lines, TGx 2002-89 GN and TGx1988-5FxTGx1989-19F-9, both in Cluster 1. In contrast, the lowest genetic distance (0.012) was observed between two IITA lines, TGx 2002-89 GN and TGx 2002-90 GN, both of which belonged to cluster 1. Within Cluster 1, the genetic distances ranged from 0.012 to 0.452 with an average of 0.337. Cluster 2 presented genetic distances ranging from 0.015 to 0.452, with an average of 0.367. For Cluster 3, the distances ranged from 0.017 to 0.433, with an average of 0.355.

The analysis of molecular variance (AMOVA) revealed that 77% of the total genetic variability was partitioned as within-population variation, which was significantly greater than the 23% partitioned among among-populations variation (Table 2). The overall genetic differentiation (PhiPT) and gene flow (Nm) for the 147 soybean genotypes were 0.233 ($p < 0.001$) and 1.649, respectively.

Table 2. Analysis of molecular variance (AMOVA), population differentiation (PhiPT), and gene flow (Nm) within and among soybean populations

Source of Variation		DF	SS	MS	Est. Var.	%
Within populations		144	184936.218	1284.279	1284.279	77

Among populations		2	34906.864	17453.432	389.395	23
Total		146	219843.082		1673.674	100
Statistical test	PhiPT				0.233	
	p (rand >= data)				0.001	
	Nm (haploid)				1.649	

DF, degrees of freedom; SS, sum of squares; MS, mean square deviation; Est.Var., estimated variance component; percentage of the total variance (%) contributed by each component and significance of variance (p-value); Nm, gene flow.

The pairwise population differentiation (PhiPT) estimates revealed that the highest degree of differentiation (0.267) was observed between populations 1 and 3, whereas the lowest degree of differentiation (0.200) occurred between populations 1 and 2. The genetic differentiation between population 2 and population 3 was 0.244. On the other hand, the pairwise population estimates of gene flow (Nm) for the three populations ranged from 1.376 to 1.998 migrants per population (Table 3).

Table 3: Pairwise population comparison using population differentiation (PhiPT), and gene flow (Nm) values based on 999 permutations from AMOVA according to Hierarchical Cluster analysis

Population 1	Population 2	PhiPT	Nm
Cluster 1	Cluster 2	0.200***	1.998
Cluster 1	Cluster 3	0.267***	1.376
Cluster 2	Cluster 3	0.244***	1.546

***; PhiPT values significantly greater than 0, $p < 0.001$

Discussion

Studying the genetic diversity of germplasm or breeding material is the best approach for understanding the existing genetic variation and effectively managing genetic resources to enhance breeding programs [73, 74]. Hence, plant breeders need such genetic analysis to execute strategic target selection and integration while maintaining significant economic traits associated with distinct crops [75].

The average value of 0.277 indicates that the markers used in this study were both informative and polymorphic. Given the bi-allelic nature of SNPs, where the PIC cannot exceed 0.5 [76], the PIC values observed in this study are suitable for differentiating the 147 soybean

accessions. Similar results have been reported in soybean studies, including Abebe et al. [2], who found a mean PIC value of 0.25 for elite soybean lines developed by IITA, and Lee et al [77], who reported a PIC value of 0.22 when evaluating 228 soybean genotypes. Tsindi et al.[78] also reported a PIC value of 0.2 for 210 South African soybean genotypes. In other self-pollinated crops, Singh et al. [76] reported a mean PIC value of 0.23 in rice. This study also demonstrated the possibility of using the selected DArTseq-SNP markers for genomic investigations in soybeans, which may serve as a foundation for future breeding efforts in the IITA soybean breeding program and conservation initiatives in Nigeria. The MAF value measures the selective ability of the marker. Owing to the bi-allelic nature of SNP markers, the MAF closest to 0.5, is best. The high average MAF value of 0.254 observed in this study indicates valuable genes can be exploited from those genotypes [75]. Compared to the results based on SNP markers reported by Hao et al. [79], our MAF values are lower. Their study revealed that the MAF ranged from 0.102 to 0.50 in soybean landraces, with an average value of 0.291. This difference might be because the materials used in the present study were advanced breeding lines, whereas Hao et al. [79] focused mainly on landraces. The average expected heterozygosity (He) of 0.344 indicates high genetic diversity within the soybean accessions, which can be effectively used for soybean improvement [75]. The Shannon-Wiever diversity index (H′), which quantifies the entropy or uncertainty in the genetic composition of a population, has a mean value of 8.597. This relatively high value indicates a genetically diverse population with various alleles and genotypes. Such a result suggests that our soybean genotypes are built on a genetic foundation, with a broad range of genetic types contributing to its overall diversity. Similarly, the inverse Simpson index (1/D), with its high mean value of 5349.145, reinforces the genetic diversity within our soybean population. It also implies a balanced distribution of genotypes without any single genotype dominating, reflecting a well-represented mix of genetic types. Comparable findings have been reported in studies on soybean [80, 81], maize [82], rice [83], and wheat [84]. Furthermore, the alpha diversity index (A), which assesses both the richness (the number of distinct genotypes or alleles) and evenness (the relative abundance of each genotype/allele) within a population, also exhibits a high mean value (4950.720). This suggests that while genetic diversity may vary across different subpopulations or sites, the overall population remains genetically rich, as reported by Adejumobi et al [85].

Analyzing population structure via SNP markers offers helpful information for preserving and tracking the genetic diversity essential for an effective breeding program [86]. ADMIXTURE

and DAPC analyses were used to determine the population structure, revealing the presence of four major populations (K = 4) for the 147 soybean genotypes. However, previous studies [2] and [6] reported different ADMIXTURES results, with ΔK values of 3 and 6, respectively. Considerable levels of admixture (42.87%) were detected among the genotypes, which likely resulted from historical gene flow, breeding practices, and inherent genetic diversity within and between the soybean populations [57]. Chander et al. [6] reported similar levels of admixture in their study of 165 soybean genotypes, which primarily consisted of IITA-bred soybean varieties. In contrast to the results of the ADMIXTURE and DAPC analyses, the hierarchical cluster (HC) method classified the 147 genotypes into three major clusters, suggesting that this could represent the optimal number of genetic clusters within the soybean accessions studied [78]. These results highlight the effectiveness of SNP markers in identifying superior accessions that have the potential to enhance the genetic diversity of the soybean population [87]. Furthermore, the results emphasize the importance of the distinct pedigrees of the soybean genotypes in maintaining genetic variation, as genotypes with similar pedigrees tended to cluster together based on their SNP profiles. Similar clustering patterns, which reflect the genetic origin of the accessions, have been reported in soybean [79–88-89,] as well as in other legume species such as cowpea [90, 91] and sesame [92]. Grouping the 147 genotypes into four distinct clusters within the first two principal components accounting for more than 60% of the total genetic variation indicates a high level of variation among the genotypes across the clusters, but high relatedness within a specific cluster. These results suggest that the genotypes within a given cluster share significant genetic similarities, making them potentially valuable for enhancing the genetic diversity of soybean breeding programs through hybridization. In support of this, Bakayoko et al. [60] highlighted that genotypes within the same cluster are genetically similar and thus could play a crucial role in genetic improvement efforts.

The results from the analysis of molecular variance (AMOVA) suggest a significant level of gene flow, with 23% of the total variation attributable to differences among populations, while 77% of the variation was observed within populations. This indicates that the majority of genetic variation resides within populations, but there is still considerable variation between them, supporting the presence of gene flow. These results are further corroborated by the gene flow (Nm) value, which plays a key role in enhancing the genetic diversity and influencing genetic differentiation of plant populations and is a crucial factor influencing genetic differentiation [93]. When Nm is greater than 1, gene flow is sufficient to counteract the effects

of genetic drift. In this study, the average gene flow value (Nm = 1.649) suggests that the soybean populations are not yet significantly impacted by genetic drift. Similar findings have been reported in previous studies on soybean [9- 89– 94,95] and other crops, such as Camelina sativa [96], wheat [97], rice [14], cowpea [74– 98], and potatoes [99]. The PhiPT value (an analogue of the fixation index Fst) of 0.23 indicates a high level of genetic differentiation among populations, suggesting limited gene exchange. In general, low Fst values close to 0 suggest that subpopulations are genetically similar, with minimal divergence, whereas an Fst of 1 indicates complete genetic fixation within subpopulations [92– 100]. This analysis also revealed significant genetic differentiation between populations 1 and 2 and between populations 2 and 3. Moreover, the differentiation between populations 1 and 3 was particularly pronounced. This substantial genetic diversity among all pairwise populations highlights the considerable diversity within the soybean accession and emphasizes the effectiveness of the selected markers for future research on soybean genetic diversity [2]. Consequently, hybridizing genotypes from different populations could introduce valuable genetic variation, enhancing genetic gain through focused selection [9].

Conclusion

The soybean lines analyzed in this study exhibit high levels of polymorphism and genetic diversity, reflecting considerable genetic variability within the population. A distinct genetic structure was observed among the sub-populations, which were grouped based on their pedigree or geographic origin. The distribution of soybean genotypes across major clusters or sub-populations, as revealed by multivariate analyses, highlights the success of IITA’s plant breeding efforts in creating a diverse genetic base. This diversity has been achieved while maintaining a focus on enhancing local adaptation to various agroecological zones within soybean-growing areas of West Africa. The diverse nature of materials used in this study, suggests these materials serve as valuable sources of genetic variation. These genotypes potentially harbor contrasting parental traits and novel alleles relevant to economically significant characteristics such as yield, drought resistance, and pod shattering. These findings present an opportunity for soybean breeders to improve the efficient selection of parental lines.

Moreover, the study emphasizes the potential need to integrate exotic germplasm into breeding programs to further enrich the genetic diversity base of soybeans in the region.

Acknowledgements

We gratefully acknowledge the technical support the soybean breeding team provided in establishing the trials and leaf sample collection**.**

References

1. Shete R, Borale S, Andhale G, Girase V. Screening of soybean genotypes for pod-shattering tolerance and association of different traits with seed yield. The Pharma Innovation Journal. 2023; 12: 1548-1551.

2. Abebe AT, Kolawole AO, Unachukwu N, Chigeza G, Tefera H, Gedil M. Assessment of diversity in tropical soybean (*Glycine max* (L.) Merr.) varieties and elite breeding lines using single nucleotide polymorphism markers. Plant Genetic Resources: Characterisation and Utilisation. 2021; 19: 20-28. https://doi.org/10.1017/S1479262121000034

3. Dean F, Science H, Ranga ANG. Soyabean the Miracle Golden Bean in Indian Foods. Acta Scientific Nutritional Health. 2019; 3: 44-49.

4. Tolorunse KD, Joseph EC, Gana AS, Azuh VO. Molecular characterization of soybean (*Glycine max* (L.) Merril) genotypes using SSR markers. Nigeria Journal of Plant Breeding. 2022; 1: 12-17.

5. Andrijanić Z, Nazzicari N, Šarčević H, Sudarić A, Annicchiarico P, Pejić I. Genetic Diversity and Population Structure of European Soybean Germplasm Revealed by Single Nucleotide Polymorphism. Plants. 2023; 12: 1837. https://doi.org/10.3390/plants12091837

6. Chander S, Lu A, Gedil M, Shah T, Otusanya GO, Asiedu R, et al. Genetic Diversity and Population Structure of Soybean Lines Adapted to Sub-Saharan Africa Using Single Nucleotide (SNP) Markers. Agronomy. 2021; 11: 604. https://doi.org/10.3390/agronomy11030604

7. Ronner E, Frankea AC, Vanlauwec B, Dianda M, Edehe E, Ukeme B, et al. Understanding

variability in soybean yield and response to P-fertilizer and rhizobium inoculants on farmers' fields in northern Nigeria. Field Crops Research. 2016; 186: 133-145. https://doi.org/10.1016/j.fcr.2015.10.023

8. Khojely DM, Ibrahim SE, Sapey E, Han T. History, current status, and prospects of soybean production and research in sub-Saharan Africa. The Crop Journal. 2018; 6: 226-235. https://doi.org/https://doi.org/10.1016/j.cj.2018.03.006

9. Shaibu AS, Ibrahim H, Miko ZL, Mohammed IB, Mohammed SG, Yusuf HL, et al. Assessment of the Genetic Structure and Diversity of Soybean and Single Nucleotide Polymorphism Markers. Plants. 2022; 11: 68. https:// doi.org/10.3390/plants11010068

10. Bhandari HR, Bhanu NA, Srivastava K, Singh MN, Shreya, Hemantaranjan A. Assessment of Genetic Diversity in Crop Plants - an overview. Adv Plants Agric Res. 2017; 7: 279-286. https://doi.org/10.15406/apar.2017.07.00255

11. Zatybekov A, Yermagambetova M, Genievskaya Y, Didorenko S, Growing P. Genetic Diversity Analysis of Soybean Collection Using Simple Sequence Repeat Markers. Plants. 2023; 12: 3445. https://doi.org/10.3390/plants12193445

12. Onda Y, Mochida K. Exploring Genetic Diversity in Plants Using High-Throughput Sequencing Techniques. Current Genomics. 2016; 17: 358–367. https://doi.org/10.2174/1389202917666160331202742

13. Schaal BA, Hayworth DA, Olsen KM, Rauscher JT, Smith WA. Phylogeographic Studies in Plants. Molecular Ecology. 1998; 7: 465–474.

14. Adeboye KA, Oyedeji OE, Alqudah AM, Börne A, Oduwaye O, Adebambo O, et al. Genetic structure and diversity of upland rice germplasm using diversity array technology (DArT)-based single nucleotide polymorphism (SNP) markers. Plant Genetic Resources: Characterisation and Utilisation. 2020; 18: 343–350. https://doi.org/10.1017/S1479262120000362

15. Liu Y, Lai Y, Wang C, Li X, Liu S, He C. Fine mapping and candidate gene analysis of qRT9, a novel major QTL with pleiotropism for yield-related traits in soybean. BMC Genomics. 2018; 19: 217.

16. Bressan EA, Neto TB, Zucchi MI, Rabello RJ, Veasey EA. Genetic structure and diversity in the *Dioscorea cayenensis*/*D. rotundata* complex revealed by morphological and

isozyme markers. Genet. Mol. Res., 2014, 13: 425–437. https://doi.org/10.4238/2014.January.21.10

17. Bressan EA, Neto TB, Zucchi MI, Rabello RJ, Veasey EA. Morphological variation and isozyme diversity in *Dioscorea alata* L. landraces from Vale do Ribeira, Brazil. Sci. Agric. 2011; 68: 494–502. https://doi.org/10.1590/S0103-90162011000400016

18. Zannou A, Agbicodo E, Zoundjihékpon J, Struik PC, Ahanchédé A, et al. Genetic variability in yam cultivars from the guinea- Sudan zone of Benin assessed by random amplified polymorphic DNA. Afr. J. Biotechnol. 2009; 8: 26–36. https://doi.org/10.5897/AJB2009.000-9005

19. Terauchi R, Chikaleke VA, Thottappilly G, Hahn SK. Origin and phylogeny of Guinea yams as revealed by RFLP analysis of chloroplast DNA and nuclear ribosomal DNA. Theor. Appl. Genet. 1992; 83: 743–751. https://doi.org/10.1007/BF00226693

20. Sonibare MA, Asiedu R, Albach DC. Genetic diversity of *Dioscorea dumetorum* (Kunth) Pax using Amplified Fragment Length Polymorphisms (AFLP) and cpDNA. Biochem. Syst. Ecol. 2010; 38: 320–334. https://doi.org/10.1016/j.bse.2010.01.012

21. Arnau G, Bhattacharjee R, Sheela MN, Chair H, Malapa R, Lebot V, et al. Understanding the genetic diversity and population structure of yam (*Dioscorea alata* L.) using microsatellite markers. PLoS ONE 2017; 12: 1–17. https://doi.org/10.1371/journal.pone.0174150

22. Mulualem T, Mekbib F, Shimelis H, Gebre E, Amelework B. Genetic diversity of yam (*Dioscorea* spp.) landrace collections from Ethiopia using simple sequence repeat markers. Aust. J. Crop Sci. 2018; 12: 1222–1230. https://doi.org/10.21475/ajcs.18.12.08.PNE885

23. Silva DM, Siqueira MVBM, Carrasco NF, Mantello CC, Nascimento WF, Veasey EA. Genetic diversity among air yam (*Dioscorea bulbifera*) varieties based on single sequence repeat markers. Genet. Mol. Res. 2016; 15: 1–12. https://doi.org/10.4238/gmr.15027929

24. Otto, E.; Anokye, M.; Asare, P.A.; Tetteh, J.P. Molecular Categorization of some Water Yam (*Dioscorea alata* L.) Germplasm in Ghana using Microsatellite (SSR) Markers. *J. Agric. Sci.* **2015**, 7(10).

25. Siqueira MV, Dequigiovanni G, Corazon-Guivin MA, Feltran JC, Veasey EA. DNA fingerprinting of water yam (*Dioscorea alata*) cultivars in Brazil based on microsatellite markers. Hortic. Bras. 2012; 30: 653–659. https://doi.org/10.1590/s0102-05362012000400015

26. Wang M, Li RZ, Yang WM, Du WJ. Assessing the genetic diversity of cultivars and wild soybeans using SSR markers. Afr J Biotechnol., 2010; 9:4857-4866.
27. Guan R, Chang R, Li Y, Wang L, Liu Z, Qiu L. Genetic diversity comparison between Chinese and Japanese soybeans (*Glycine max* (L.) Merr.) revealed by nuclear SSRs. Genet Resour Crop Evol., 2010; 57:229-242
28. Kujane K, Sedibe MM, Mofokeng A. Genetic diversity analysis of soybean (Glycine max (L.) Merr.) genotypes making use of SSR markers. Australian Journal of Crop Science, 2019; 13: 1113–1119. https://doi.org/10.21475/ajcs.19.13.07.p1638
29. Acinas SG, Sarma-Rupavtarm R, Klepac-Ceraj V, Polz MF. PCR-induced sequence artefacts and bias: Insights from comparison of two 16s rRNA clone libraries constructed from the same sample. Appl. Env. Microb. 2005; 71: 8966–8969. https://doi.org/10.1128/AEM.71.12.8966-8969.2005
30. Brakenhoff RH, Schoenmakers JGG, Lubsen NH. Chimeric cDNA clones: A novel PCR artefact. Nucleic Acids Res. 1991; 19: 1949. https://doi.org/10.1093/nar/19.8.1949
31. Cline J, Braman JC, Hogrefe HH. PCR fidelity of Pfu DNA polymerase and other thermostable DNA polymerases. Nucleic Acids Res. 1996; 24: 3546–3551. https://doi.org/10.1093/nar/24.18.3546
32. Kulibaba RA, Liashenko YV. Influence of the PCR artefacts on the genotyping efficiency by the microsatellite loci using native polyacrylamide gel electrophoresis. Cytol. Genet. 2016; 50: 162–167. https://doi.org/10.3103/S0095452716030087
33. Tsykun T, Rellstab C, Dutech C, Sipos G, Prospero S. Comparative assessment of SSR and SNP markers for inferring the population genetic structure of the common fungus *Armillaria cepistipes*. Heredity 2017; 119: 371–380. https://doi.org/10.1038/hdy.2017.48
34. Yu Z, Fredua-Agyema R, Hwang SF, Strelkov SE. Molecular genetic diversity and population structure analyses of rutabaga accessions from Nordic countries as revealed by single nucleotide polymorphism markers. BMC Genomics 2021; 22: 1–13. https://doi.org/10.1186/s12864-021-07762-4
35. Vivodík M, Balážová Ž, Chňapek M, Hromadová Z, Mikolášová L, Gálová Z. Molecular Characterization and Genetic Diversity Studies of Soybean (*Glycine max* L.) Cultivars Using Rapid Markers. Journal of Microbiology, Biotechnology and Food Sciences, 2022; 12: 1–5. https://doi.org/10.55251/jmbfs.9219

36. Vignal A, Milan D, SanCristobal M, Eggen A. A review on SNP and other types of molecular markers and their use in animals. Genetics 2002; *34*: 275–305. https://doi.org/10.1051/gse

37. Verma S, Gupta S, Bandhiwal N, Kumar T, Bharadwaj C, Bhatia, S. High-density linkage map construction and mapping of seed trait QTLs in chickpea (*Cicer arietinum* L.) using genotyping-by-sequencing (GBS). Sci. Rep., 2015; 5: 17512.

38. Fischer MC, Rellstab C, Leuzinger M, Roumet M, Gugerli F, Shimizu KK, et al. Estimating genomic diversity and population differentiation - an empirical comparison of microsatellite and SNP variation in Arabidopsis halleri. BMC Genomics. 2017; 18: 1–15. https://doi.org/10.1186/s12864-016-3459-7

39. Lam H-M, Xu X, Liu X, Chen W, Yang G, Wong F-L, et al. Resequencing of 31 wild and cultivated soybean genomes identifies patterns of genetic diversity and selection. Nature Genetics. 2010; 42: 1053–1059. https://doi.org/10.1038/ng.715

40. Zhou Z, Jiang Y, Wang Z, Gou Z, Lyu J, Li W, et al. Resequencing 302 wild and cultivated accessions identifies genes related to domestication and improvement in soybean. Nature Biotechnology. 2015; 33: 408–414. https://doi.org/10.1038/nbt.3096

41. dos Santos JVM, Valliyodan B, Joshi T, Khan SM, Liu Y, Wang J, et al. Evaluation of genetic variation among Brazilian soybean cultivars through genome resequencing. BMC Genomics. 2016; 17: 1–18. https://doi.org/10.1186/s12864-016-2431-x

42. Valliyodan B, Brown AV, Wang J, Patil G, Liu Y, Otyama PI, et al. Genetic variation among 481 diverse soybean accessions, inferred from genomic re-sequencing. Scientific Data. 2021; .8: 1–9. https://doi.org/10.1038/s41597-021-00834-w

43. Torkamaneh D, Laroche J, Valliyodan B, O’Donoughue L, Cober E, Rajcan I, et al.. Soybean (Glycine max) Haplotype Map (GmHapMap): a universal resource for soybean translational and functional genomics. Plant Biotechnology Journal. 2021; 19: 324–334. https://doi.org/10.1111/pbi.13466

44. LeeY, Woo DU, Kang YJ. SoyDbean: a database for SNPs reconciliation by multiple versions of soybean reference genomes. Scientific Reports. 2023; 13: 1–10. https://doi.org/10.1038/s41598-023-42898-1

45. Yang X, Ren R, Ray R, Xu J, Li P, Zhang M, et al. Genetic diversity and population structure of core watermelon (*Citrullus lanatus*) genotypes using DArTseq-based SNPs.

Plant Genetic Resources: Characterisation and Utilisation. 2016; 14: 226–233. https://doi.org/10.1017/S1479262115000659

46. Kilian A, Wenzl P, Huttner E, Carling J, Xia L, Blois H, et al. Diversity arrays technology: a generic genome profiling technology on open platforms. Methods in Molecular Biology (Clifton, N.J.). 2012; 888: 67–89. https://doi.org/10.1007/978-1-61779-870-2_5

47. Chiemeke FK, Olasanmi B, Agre PA, Mushoriwa H, Chigeza G, Abebe AT. Genetic Diversity and Population Structure Analysis of Soybean [*Glycine max* (L.) Merrill] Genotypes Using Agro Morphological Traits and SNP Markers. Genes. 2024;15: 373. https://doi.org/10.3390/ genes15111373

48. Czembor E, Czembor JH, Suchecki R, Watson-Haigh NS. DArT-based evaluation of soybean germplasm from Polish Gene Bank. BMC Research Notes. 2021; 14: 1–6. https://doi.org/10.1186/s13104-021-05750-1

49. Tomkowiak A, Nowak B, Sobiech A, Bocianowski J, Wolko Ł, Spychała J. The Use of DArTseq Technology to Identify New SNP and SilicoDArT Markers Related to the Yield-Related Traits Components in Maize. Genes, 2022; 13: 848. https://doi.org/10.3390/genes13050848

50. Khodadadi M, Fotokian MH, Miransari M. Genetic diversity of wheat (*Triticum aestivum* L.) genotypes based on cluster and principal component analyses for breeding strategies. Aust. J. Crop Sci. 2011, 5, 17–24.

51. Sohail Q, Manickavelu A, Ban T. Genetic diversity analysis of Afghan wheat landraces (*Triticum aestivum*) using DArT markers. Genet. Resour. Crop Evol. 2015; 62: 1147–1157.

52. Adu BG, Adu Amoah R, Aboagye LM, Abdoul Aziz MG, Boampong R. High-Density DArT Markers and Phenotypic Characterization of Cowpea Accessions (*Vigna unguiculata* (L.) Walp). Advances in Agriculture.2021; ID 6336684, 12p. https://doi.org/10.1155/2021/6336684

53. Gbedevi KM, Boukar O, Ishikawa H, Abe A, Ongom PO. Genetic Diversity and Population Structure of Cowpea [*Vigna unguiculata* (L .) Walp .] Germplasm Collected from Togo Based on DArT Markers. Genes. 2021; 12: 1451.https://doi.org/10.3390/genes12091451

54. Mace ES, Xia L, Jordan DR, Halloran K, Parh DK, Huttner E, et al. DArT markers:

Diversity analyses and mapping in Sorghum bicolor. BMC Genom. 2008; 11: 1–11. doi:10.1186/1471-2164-9-26

55. Egea LA, Mérida-garcía R, Kilian A, Hernandez P, Dorado G. Assessment of Genetic Diversity and Structure of Large Garlic (*Allium sativum*) Germplasm Bank , by Diversity Arrays Technology Platform (DArTseq). Front. Genet. 2017; 8: 1–9. https://doi.org/10.3389/fgene.2017.00098

56. Alemu A, Feyissa T, Letta T, Abeyo B. Genetic diversity and population structure analysis based on the high-density SNP markers in Ethiopian durum wheat (*Triticum turgidum* ssp. durum). BMC Genet. 2020; 21:18. doi: 10.1186/s12863-020-0825-x.

57. Pritchard JK, Stephens M, Donnelly P. Inference of population structure using multilocus genotype data. Genetics. 2000; 155:945–959. https://doi.org/10.1093/genetics/155.2.945

58 Odong TL, Jansen J, Van Eeuwijk FA, van Hintum TJ. Quality of core collections for effective utilisation of genetic resources review, discussion and interpretation. Theoretical and Applied Genetics, 2013; 126: 289-305.

59. Schmutz J, Cannon SB, Schlueter J, Ma J, Mitros T, Nelson W, et al.. Genome sequence of the palaeopolyploid soybean. Nature. 2010; 463: 178–183. https://doi.org/10.1038/nature08670

60. Bakayoko L, N'Da Pokou D, Kouassi AB, Agre PA, Kouakou AM, Dibi KE, et al. Diversity Of Water Yam (*Dioscorea Alata* L.) Accessions from Côte D'ivoire Based On SNP Markers And Agronomic Traits. Plants. 2021; 10: 1–18. https://doi.org/10.3390/plants10122562

61. Danecek P, Auton A, Abecasis G, Albers CA, Banks E, DePristo MA, et al. The variant call format and VCFtools. Bioinformatics. 2011; 27: 2156–2158. https://doi.org/10.1093/bioinformatics/btr330

62. Purcell S, Neale B, Todd-Brown K, Thomas L, Ferreira MAR, Bender D, et al. PLINK: A tool set for whole-genome association and population-based linkage analyses. American Journal of Human Genetics. 2007; 81: 559–575. https://doi.org/10.1086/519795

63. Oksanen J; Guillaume BF, Friendly M, Roeland KP, Dan ML, Minchin PR, et al. Vegan: community ecology package 2019. R package version 2.5–5. Available at https://CRAN.R-project.org/package=vegan

64. Yin L. R package “CMPlots”. 2019. Available: https://github.com/YinLiLin/RCMplot (Accessed June 2024).

65. Agre PA, Edemodu A, Obidiegwu JE, Adebola P, Asiedu R, Asfaw A. Variability and genetic merits of white Guinea yam landraces in Nigeria. Frontiers in Plant Science. 2023; 14: 1–12. https://doi.org/10.3389/fpls.2023.1051840

66. Salazar E, González M, Araya C, Mejía N, Carrasco B. Genetic diversity and intra-racial structure of Chilean Choclero corn (*Zea mays* L.) germplasm revealed by simple sequence repeat markers (SSRs). Scientia Horticulturae. 2017; 225: 620–629. https://doi.org/10.1016/j.scienta.2017.08.006

67. Jombart T. Adegenet: A R package for the multivariate analysis of genetic markers. Bioinformatics. 2008; 24: 1403–1405. https://doi.org/10.1093/bioinformatics/btn129

68. Drost HG. Philentropy: Information Theory and Distance Quantification with R. Journal of Open Source Software. 2018; 3: 765. https://doi.org/10.21105/joss.00765

69. Paradis E, Claude J, Strimmer K. APE : Analyses of Phylogenetics and Evolution in R language. Bioinformatics. 2004; 20: 289–290. https://doi.org/10.1093/bioinformatics/btg412

70. Lê S, Josse J, Husson F. FactoMineR: An R Package for Multivariate Analysis. Journal of Statistical Software. 2008; 25: 1–18.

71. Kassambara A, Mundt F. Factoextra: Extract and Visualize the Results of Multivariate Data Analyses. R Package Version 1.0.7. 2020. https://CRAN.R-project.org/package=factoextra

72. Peakall R, Smouse PE. GenALEx 6.5: Genetic analysis in Excel. Population genetic software for teaching and research —an update. Bioinformatics. 2012; 28: 2537–2539. https://doi.org/10.1093/bioinformatics/bts460

73. Al-Abdallat AM, Karadsheh A, Hadadd NI, Akash MW, Ceccarelli S, Baum M, et al. Assessment of genetic diversity and yield performance in Jordanian barley (*Hordeum vulgare* L.) landraces grown under Rainfed conditions. BMC Plant Biology. 2017; 17: 1–13. https://doi.org/10.1186/s12870-017-1140-1

74. Ketema S, Tesfaye B, Keneni G, Fenta BA, Assefa E, Greliche N, et al. DArTSeq SNP-based markers revealed high genetic diversity and structured population in Ethiopian

cowpea [*Vigna unguiculata* (L.) Walp] germplasms. PLoS ONE. 2020; 15: 1–20. https://doi.org/10.1371/journal.pone.0239122

75. Yirgu M, Kebede M, Feyissa T, Lakew B, Woldeyohannes AB, Fikere M. Single nucleotide polymorphism (SNP) markers for genetic diversity and population structure study in Ethiopian barley (*Hordeum vulgare* L.) germplasm. BMC Genomic Data. 2023; 24: 1–13. https://doi.org/10.1186/s12863-023-01109-6

76. Singh N, Choudhury DR, Singh AK, Kumar S, Srinivasan K, Tyagi RK, Singh NK, Singh R. Comparison of SSR and SNP markers in estimation of genetic diversity and population structure of Indian rice varieties. PLoS ONE. 2013; 8:1–14

77. Lee YG, Jeong N, Kim JH, Lee K, Kim KH, Pirani A, et al. Development, validation and genetic analysis of a large soybean SNP genotyping array. Plant Journal. 2015;. 81: 625–636. https://doi.org/10.1111/tpj.12755

78. Tsindi A, Eleblu JSY, Gasura E, Mushoriwa H, Tongoona P, Danquah EY, et al. Analysis of population structure and genetic diversity in a Southern African soybean collection based on single nucleotide polymorphism markers. CABI Agriculture and Bioscience. 2023; 4: 1–14. https://doi.org/10.1186/s43170-023-00158-2

79. Hao D, Cheng H, Yin Z, Cui S, Zhang D, Wang H, et al. Identification of single nucleotide polymorphisms and haplotypes associated with yield and yield components in soybean (*Glycine max*) landraces across multiple environments. Theoretical and Applied Genetics. 2012; 124: 447–458. DOI 10.1007/s00122-011-1719-0

80. Liu S, Zhang L, Zhao X. Genetic Diversity of Soybean Germplasm in China and its Implications for Breeding Programs. Journal of Agricultural Science. 2017; 155: 279–291.

81. Zhang, X, Zhao Y, Li F. Genetic Diversity and Population Structure of Soybean Genotypes under Different Ecological Zones in China. Plant Breeding. 2020; 139: 664–672.

82. Li W, Liu J, Yang Y. Genetic Diversity of Maize Populations in Northern China: Insights from SSR Markers. Crop Science.2019; .59: 1120–1130.

83. Singh R, Kumar R, Sharma R. Genetic Diversity of Rice (*Oryza sativa*) Germplasm and its Relationship with Agronomic Traits. Field Crops Research, 2020;. 253, 107790.

84. Wang Y, Zhou X, Zhao L. Genetic Diversity of Wheat (Triticum aestivum) Revealed by

Microsatellite Markers and Its Implications for Breeding Programs. PLOS ONE. 2018;. 13: e0207550.

85. Adejumobi II, Agre PA, Onautshu DO, Adheka, JG. Diversity , trait preferences , management and utilization of yams landraces (*Dioscorea species*): an orphan crop in DR Congo. Scientific Reports.2022; 12: 1–16. https://doi.org/10.1038/s41598-022-06265-w

86. Amponsah AE, Esuma W, Alicai T, Bhattacharjee R, Dramadri IO, Edema R, et al. Genetic diversity and population structure of Uganda's yam (*Dioscorea* spp.) genetic resource based on DArTseq. PLoS ONE. 2023; 18: 1–16. https://doi.org/10.1371/journal.pone.027753739

87. Adewumi AS, Adejumobi II, Opoku VA, Asare PA, Adu MO., Taah KJ, et al.. Exploring quantitative trait nucleotides associated with response to yam mosaic virus severity and tuber yield traits in *Dioscorea praehensilis* Benth . association scanning. Front. Hortic. 2024; 3: 1–15. https://doi.org/10.3389/fhort.2024.1459476

88. Lee G-A, Choi Y-M, Yi J-Y, Chung J-W, Lee M-C, Ma K-H, et al. Genetic diversity and population structure of Korean soybean collection Using 75 microsatellite markers. Korean J Crop Sci. 2014; 59: 492–497. https:// doi. org/ 10. 7740/ kjcs.

89. Liu Z, Li H, Wen Z, Fan X, Li Y, Guan R, Guo Y, et al. Comparison of genetic diversity between Chinese and American soybean (*Glycine max* (L.)) accession revealed by high-density SNPs. Front. Plant Sci. 2017. https:/ doi.org/ 10.3389/fpls.2017.02014.

90. Fatokun C, Girma G, Abberton M, Gedil M, Unachukwu N, Oyatomi O, et al. Genetic diversity and population structure of a mini-core subset from the world cowpea (*Vigna unguiculata* (L.) Walp.) germplasm collection. Sci Rep 2018; 8:1–10. https:// doi. org/ 10. 1038/ s41598- 018- 34555-9.

91. Sodedji FAK, Agbahoungba S, Agoyi EE, Kafoutchoni MK, Choi J, Nguetta SPA, Diversity, population structure, and linkage disequilibrium among cowpea accessions. Plant Genome. 2021. https:// doi. org/ 10. 1002/ tpg2. 20113.

92. Basak M, Uzun B, Yol E. Genetic diversity and population structure of the Mediterranean sesame core collection with use of genome-wide SNPs developed by double digest RAD Seq. PLoS ONE. 2019;14:1–15. https:// doi. org/ 10. 1371/ journal. pone. 02237 57.

93. Weising K, Nybom H, Wolff K, Kahl G. DNA Fingerprinting in Plants. CRC Press: Boca Raton, FL, USA; 2005: ISBN 978-0-8493-1488-9

94. Jeong SC, Moon JK, Park SK, Kim MS, Lee K, Lee SR, et al. Genetic diversity patterns and domestication origin of soybean. Theoretical and Applied Genetics. 2019; 132: 1179–1193. https://doi.org/10.1007/s00122-018-3271-7

95. Lukanda MM, Dramadri OI, Amponsah AE, Arusei P, Gitonga WH; Wasswa P, et al. Genetic diversity and population structure of Ugandan soybean (*Glycine max* (L.) germplasm based on DArTseq. Plant Molecular Biology Reporter. 2023; 41: 417-426. https://doi.org/10.1007/s11105-023-01375–9c

96. Luo Z, Brock J, Dyer JM, Kutchan T, Schachtman D, Augustin M, et al. Genetic diversity and population structure of a *Camelina sativa* spring panel. Frontiers in Plant Science. 2019; 10: 1–12. https://doi.org/10.3389/fpls.2019.00184

97. Eltaher S, Sallam A, Belamkar V, Emara HA, Nower AA, Salem KF, et al. Genetic diversity and population structure of F3:6 Nebraska Winter wheat genotypes using genotyping-by-sequencing. Frontiers in Genetics. 2018; 9: 1–9. https://doi.org/10.3389/fgene.2018.00076

98. Gomes AMF, Draper D, Talhinhas P, Batista Santos P, Simões F, Nhantumbo N, et al. Genetic diversity among cowpea (*Vigna unguiculata* (L.) Walp.) landraces suggests central Mozambique as an important hotspot of variation. Agronomy. 2020; 10: 1893. https://doi.org/10.3390/agronomy10121893

99. Lee KJ, Sebastin R, Cho GT, Yoon M, Lee GA, Hyun DY. Genetic Diversity and Population Structure of Potato Germplasm in RDA-Genebank: Utilization for Breeding and Conservation. Plants. 2021; 10: 752. https://doi.org/10.3390/ plants10040752

100. Mohammadi SA, Prasanna BM. Analysis of genetic diversity in crop plants— salient statistical tools and considerations. Crop Sci. 2003; 43:1235–48. https:// doi. org/ 10. 2135/ crops ci2003. 1235.

Supporting information:

S1 Table. List of the soybean (*Glycine max* (L.) Merril) accessions used in this study and their respective origins. (DOCX)

S1 Fig. Summary statistics of 7,083 single nucleotide polymorphism (SNP) markers used for genotyping 147 soybean accessions. (a) Expected heterozygosity, (b) observed heterozygosity, (c) minor allele frequency and (d) polymorphic information content. (JPG)

S2 Fig. Graph showing the best k value via Bayesian information criterion analysis. (JPG)

S3 Fig. Silhouette graph showing the optimal number of hierarchical clusters. (JPG)

Author contributions:

Conceptualization: Tenena Silue, Paterne Angelot Agre, Bunmi Olasanmi, Abush Tesfaye Abebe.

Data curation: Tenena Silue.

Formal analysis: Tenena Silue, Adeyinka Saburi Adewumi, Paterne Angelot Agre.

Investigation: Tenena Silue

Methodology: Tenena Silue, Paterne Angelot Agre, Adeyinka Saburi Adewumi, Abush Tesfaye Abebe.

Resources: Abush Tesfaye Abebe

Software: Tenena Silue, Adeyinka Saburi Adewumi, Paterne Angelot Agre.

Supervision: Paterne Angelot Agre, Bunmi Olasanmi, Abush Tesfaye Abebe.

Validation: Tenena Silue, Adeyinka Adewumi, Paterne Angelot Agre, Abush Tesfaye Abebe.

Visualization: Tenena Silue, Adeyinka Adewumi, Paterne Angelot Agre.

Writing – original draft: Tenena Silue

Writing – review & editing: Tenena Silue, Adeyinka Saburi Adewumi, Idris Ishola Adejumobi, Paterne Angelot Agre, Bunmi Olasanmi, Abush Tesfaye Abebe.

Click here to access/download;Figure;Figure 1 PONE.tif

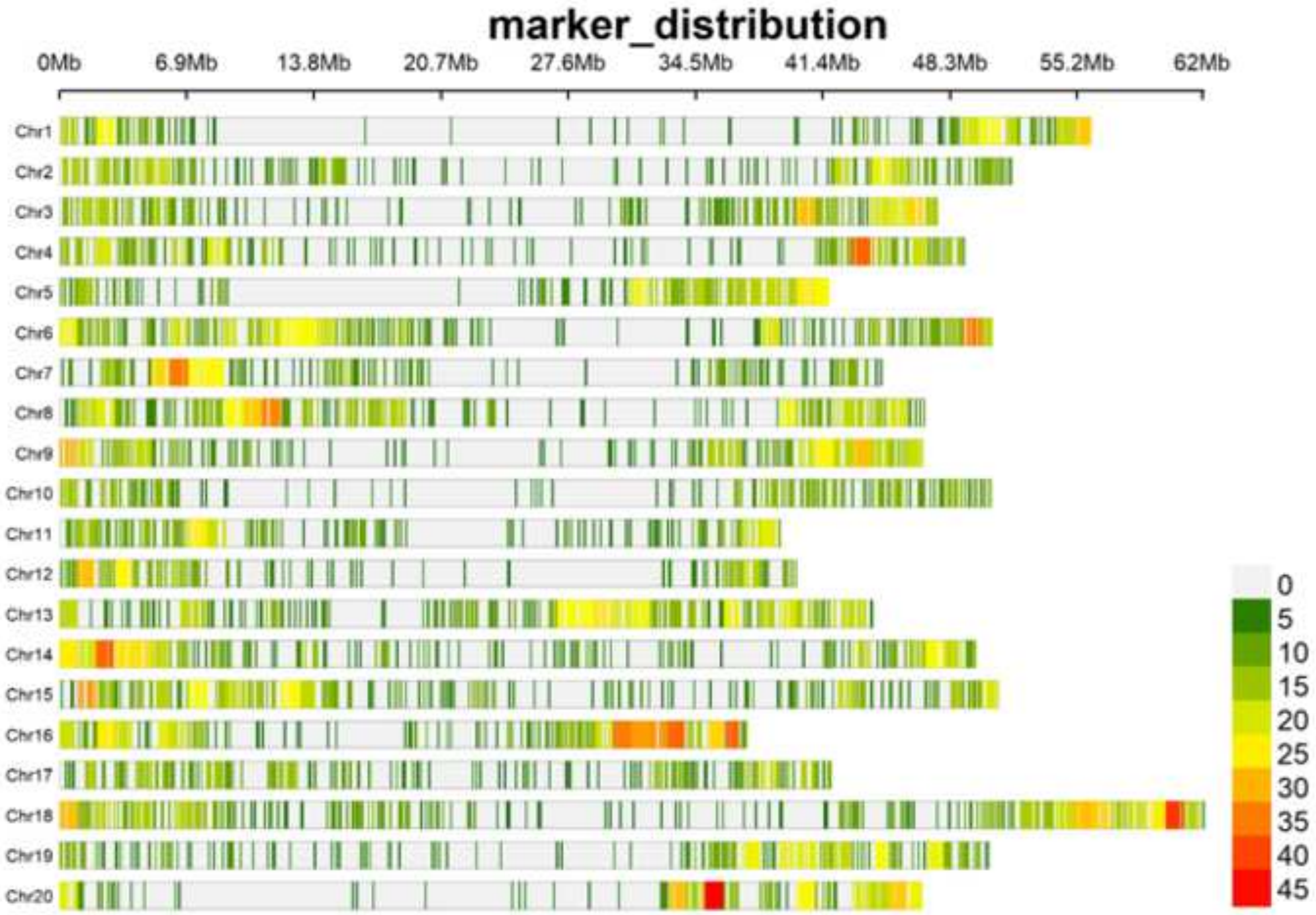

Click here to access/download;Figure;Figure 2 PONE.tif

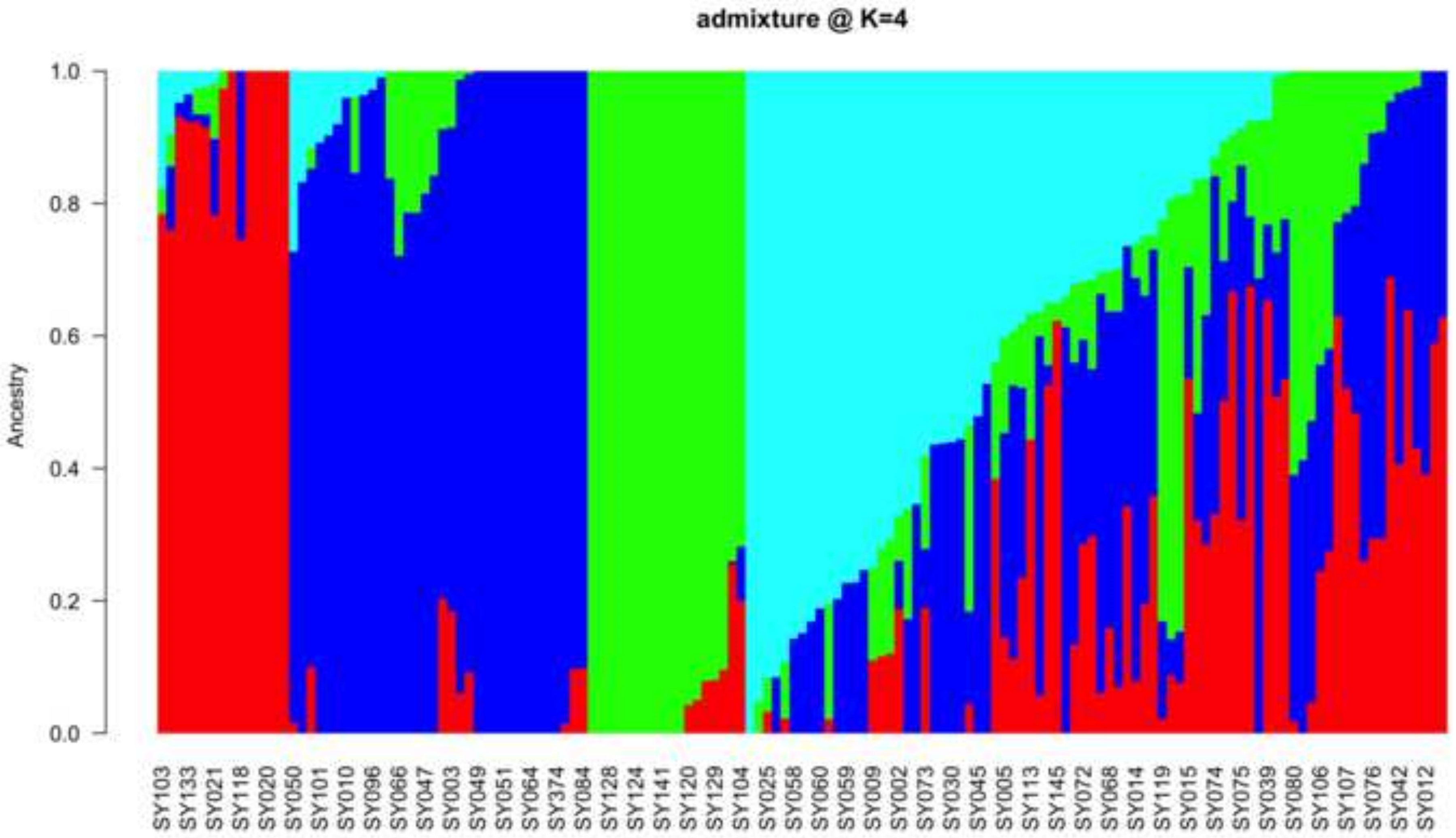

Click here to access/download;Figure;Figure 3 PONE.tif

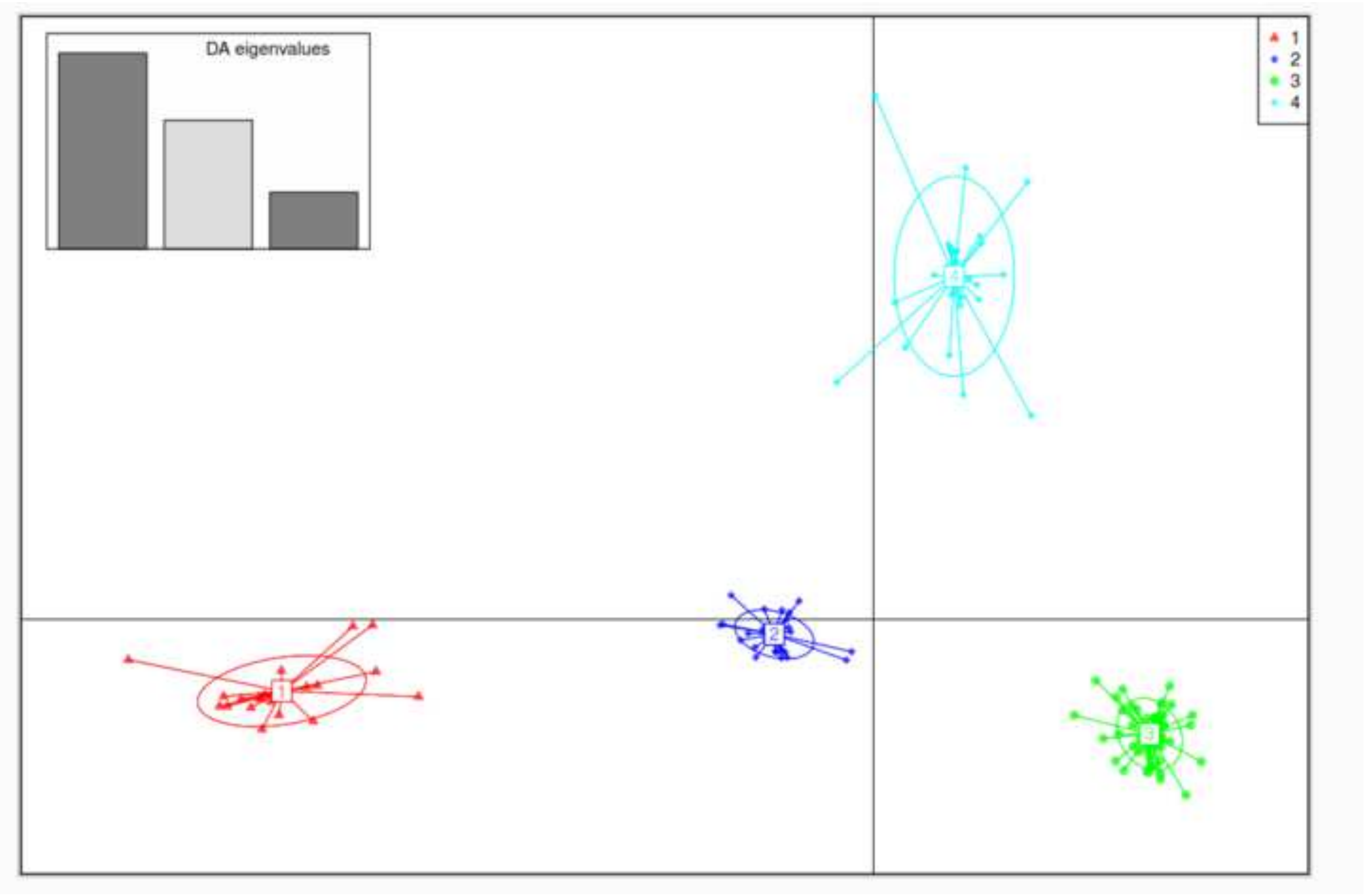

Click here to access/download;Figure;Figure 4 PONE.tif

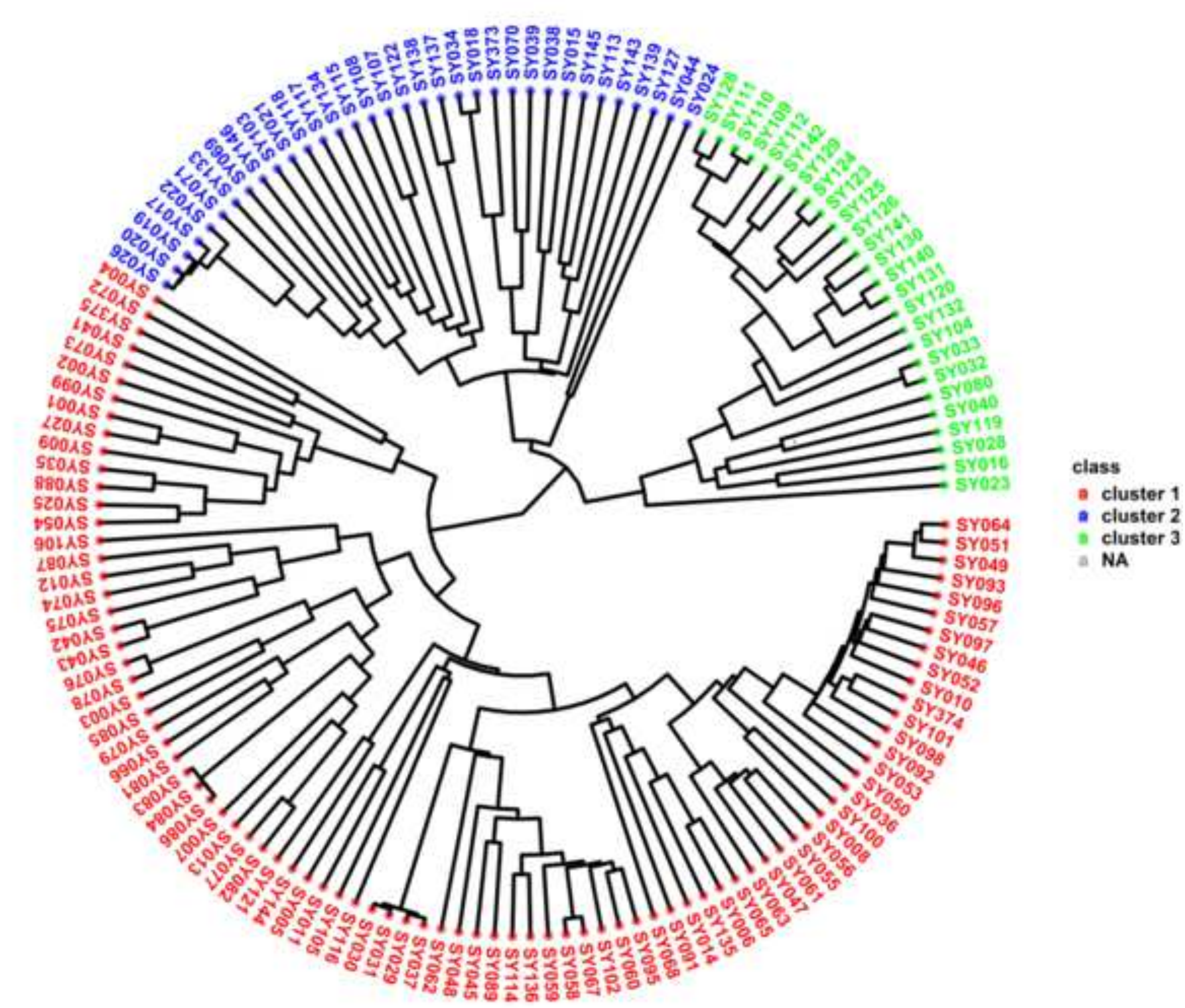

Click here to access/download;Figure;Figure 5 PONE.tif

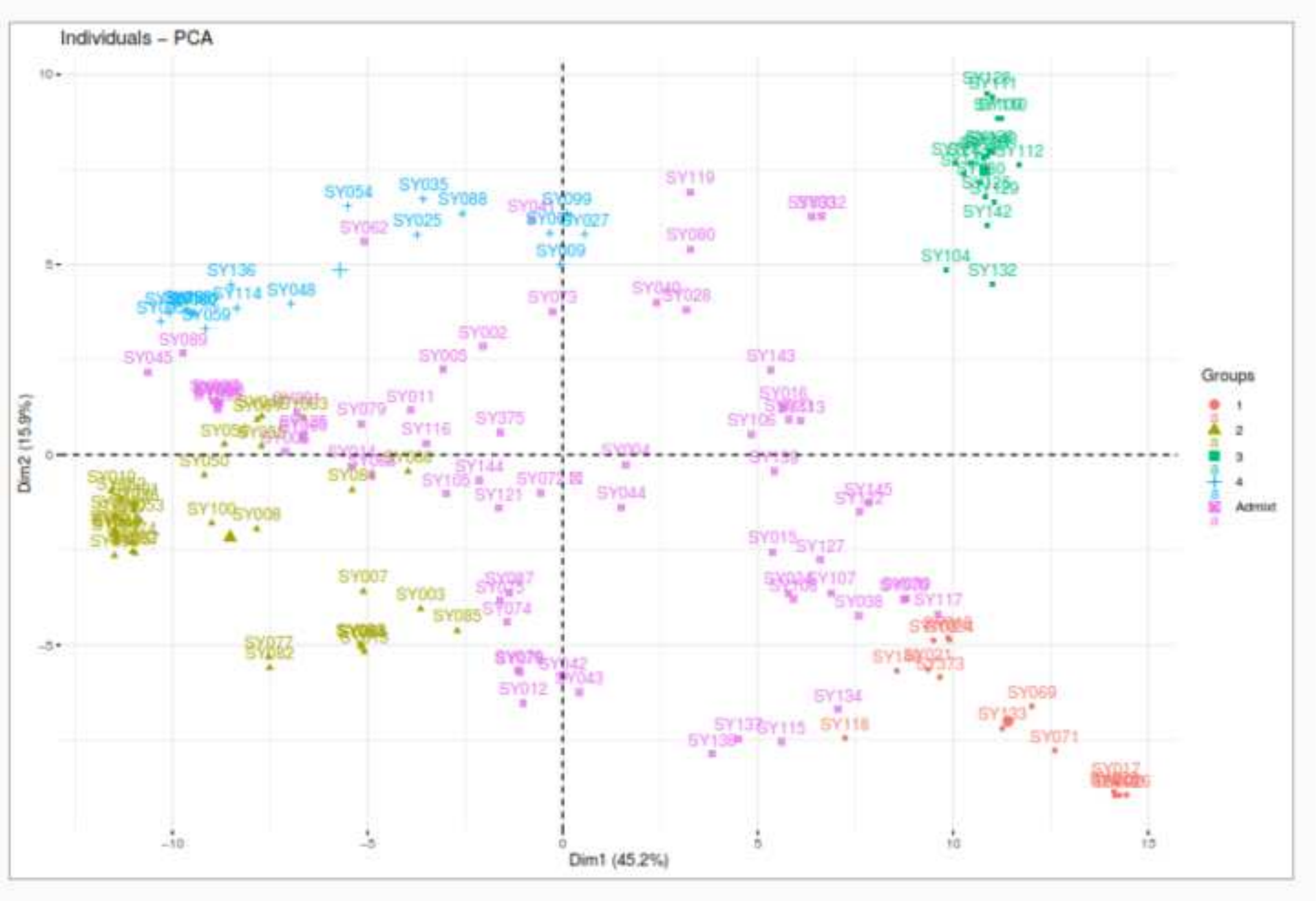

Click here to access/download
Supporting Information - Compressed/ZIP File Archive
Supporting informations.zip

RESEARCH ARTICLE

Genetic diversity and population structure of soybean (*Glycine max* (L.) Merril) germplasm.

Tenena Silue[1,2,*], Paterne Angelot Agre[2], Bunmi Olasanmi[3], Adeyinka Saburi Adewumi[2], Idris Ishola Adejumobi[2] and Abush Tesfaye Abebe [2,*]

[1] Pan African University Life and Earth Sciences Institute (including Health and Agriculture), University of Ibadan, Ibadan, Oyo State, Nigeria

[2] International Institute of Tropical Agriculture (IITA), Ibadan, Oyo State, Nigeria

[3] Department of Crop and Horticultural Sciences, University of Ibadan, Ibadan, Oyo State, Nigeria

* Corresponding authors

Email: at.abebe@cgiar.org, siluetenenan@yahoo.fr

Abstract

Soybean (*Glycine max* (L.) Merril) is a significant legume crop for oil and protein. However, its yield in Africa is less than half the global average resulting in low production, which is inadequate for satisfying the continent's needs. To address this disparity in productivity, it is crucial to develop new high-yielding cultivars by utilizing the genetic diversity of existing germplasms. Consequently, the genetic diversity and population structure of various soybean accessions were evaluated in this study. To achieve this objective, a collection of 147 soybean accessions was genotyped using the Diversity Array Technology Sequencing method, enabling high-throughput analysis of 7,083 high-quality single-nucleotide polymorphisms (SNPs) distributed across the soybean genome. The average values observed

for polymorphism information content (PIC), minor allele frequency, expected heterozygosity and observed heterozygosity were 0.277, 0.254, 0.344, and 0.110, respectively. The soybean genotypes were categorized into four groups on the basis of model-based population structure, principal component analysis, and discriminant analysis of the principal component. Alternatively, hierarchical clustering was used to organize the accessions into three distinct clusters. Analysis of molecular variance indicated that the genetic variance (77%) within the populations exceeded the variance (23%) among them. The insights gained from this study will assist breeders in selecting parental lines for genetic recombination. The present study demonstrates that soybean improvement is viable within the IITA breeding program, and its outcome will help to optimize the genetic enhancement of soybeans.

Keywords: DArTSeq, SNPs, DAPC, admixture, AMOVA

Introduction

Soybean (*Glycine max* (L.) Merril) is a self-pollinated crop from the Fabaceae family with a diploid chromosome number of 2n = 40 [1]. It is one of the world's major legumes and oil crops in terms of production and trade [2]. Soybean contains approximately 38–42% high-quality protein and 18–20% oil rich in essential fatty acids [3]. In Nigeria, soybean is used to produce nutritious drinks known as “soymilk” and “awara” (soybean cake). It is also a crucial ingredient in poultry and fish feed and is also used in infant meals. The oil is utilized in cooking and as a base for mayonnaise, margarine, salad dressings, and shortening [4].

Globally, soybean cultivation covers approximately 121 million hectares, with an estimated total production of 334 million tons annually [5]. The top three producers (Brazil, the United States, and Argentina) together contribute 73% of the world’s production. In Africa, approximately 2.55 million hectares are dedicated to soybean cultivation, with an average productivity of 1,348 kg per hectare. South Africa, Nigeria, and Zambia are the leading

producers on the continent, with annual production rates of 1.32, 0.73, and 0.35 million tons, respectively [6].

Soybean cultivation in Africa typically yields less than 1.5 t. ha^{-1}, which is significantly below the potential yield of over 3 t. ha^{-1} [7]. This low productivity might be attributed to various factors, including the limited availability of high-yielding and climatically resilient improved varieties, poor soil fertility, diseases and pests, high pod shattering, inadequate agronomic practices, and particularly drought caused by inconsistent rainfall [8]. Therefore, there is a need for improved soybean varieties that are resilient to these biotic and abiotic stresses [9]. Furthermore, genetic diversity in many crops has decreased over time as commercial plant breeding focuses on enhancing one or a few traits and/or uses a limited number of exceptional genotypes to create a breeding population [10].

Exploiting and conserving crop genetic diversity is essential for developing new cultivars with desirable traits [11]. Assessing genetic diversity within germplasm is essential for expanding a core collection and enhancing germplasm utilization in breeding programs [2]. Identifying genes regulating beneficial traits in existing populations such as breeding lines, cultivars, landraces, and wild relatives, is crucial for improving current varieties and promoting sustainable farming practices [12]. Additionally, understanding genetic variability within and between plant populations aids breeders in improving breeding strategies- [13].

Crop variability can be assessed at both the phenotypic and genotypic levels using statistical methods to separate genetic and environmental components [14]. While morphological markers detect diversity, they are less effective than DNA markers because of their subjectivity, limited number, and environmental sensitivity [9]. DNA markers are more effective for evaluating genetic diversity, aiding in the efficient use of germplasms for conservation and crop yield improvement. Soybean genetic diversity has been assessed using various biochemical and molecular markers [15], including isozymes [16, 17], random amplified

Formatted: Indent: First line: 0"

polymorphic DNA (RAPD) [18], random fragment length polymorphism (RFLP) [19], amplified fragment length polymorphism (AFLP) [20], simple sequence repeats (SSR) [21, 25], and single nucleotide polymorphisms (SNPs). Among these, SSR markers are effective for identifying genetic relationships within soybean populations [26, 28], polymerase chain reaction (PCR) based amplification can sometimes result in sequence artefacts, complicating genotyping [29, 34]. RAPD markers, although useful, have limitations like low discriminatory power and high genotyping costs [35]. The rise of next-generation sequencing (NGS) has made SNP markers the preferred choice for studying genetic diversity due to their precision, cost-effectiveness, and even distribution across the genome [36, 37].

According to Fischer et al. [38], SNP markers are the most effective among the molecular markers used as genomic resources for identifying variations in crop varieties, including soybeans. The high abundance in the genome and the ability to identify variation at a single locus make SNP markers outstanding among the marker groups explored for genomic activity [38]. -Studies have generated extensive SNP data to explore genetic diversity between wild and cultivated soybeans, focusing on wild soybean allele diversity [39], uncovering valuable genetic information for breeding efforts [40], examining the genetic diversity and structure [41,42], and creating detailed haplotype maps using whole genome sequencing [43, 44].

Formatted: English (United States)

Diversity Array Technology (DArT) is a high-throughput genotyping method that provides cost-effective, scalable, whole-genome profiling, making it a versatile tool for various genetic applications [45]. It offers better coverage and fewer missing data compared to other next-generation sequencing (NGS) platforms and has been successfully used in crops like soybean [9–47, 48], maize [49], wheat [50, 51], cowpea and [52, 53], sorghum [54], and garlic [55].

SNP markers, which are abundant and stable across the genome, are ideal for studying genetic variation and population structure [56]. While SNPs have been widely used to assess

genetic diversity in soybeans globally, there is limited research on African germplasm, especially IITA's breeding materials. Understanding the genetic diversity of IITA's soybean germplasm can reveal distinct sub-populations, and historical breeding patterns, and guide future breeding efforts [57, 58]. Population structure analysis is crucial for avoiding inbreeding, optimizing parent selection, and enhancing breeding outcomes. This study uniquely explores the genetic diversity of IITA's soybean germplasm using SNP markers, filling a critical gap in research on African soybean breeding populations.

Given the importance of genetic diversity assessment in optimizing breeding strategy for the IITA soybean improvement and the utility of SNP markers for improved precision for genetic diversity assessment, this study aims to assess the genetic diversity and population structure of IITA soybean germplasm using SNP markers. This will provide valuable insights for enhancing soybean breeding programs in SSA and contribute to the broader goal of improving food security in the region.

Materials and methods

Plant materials, planting, and leaf sampling and DNA extraction procedure

A total of 147 soybean accessions, comprised of 130 genotypes from the IITA soybean breeding program, and 17 genotypes sourced from the United States Department of Agriculture (USDA) genetic resource center (14 genotypes), Ghana (1 genotype), Uganda (1 genotype), and a private seed company (SeedCo) (1 genotype) (list of germplasms, S1 Table), were selected and utilized for a molecular-based diversity assessment.

The 147 soybean accessions were sown and grown to the seedling stage in a screen house at IITA station Ibadan, Nigeria at 243 m.a.s., 7°30′8″N longitude and 3°54′37″E latitude. Three weeks after planting, five-leaf discs 5 mm in diameter from young and healthy leaves

were collected via a biopsy curette from the leaf blades of each of the 147 genotypes. The leaf samples were placed into 96-well collection plates (12 x 8-strip tubes per 96-deep well plate) and lyophilized using a Labconco Freezone 6 plus dryer. The lyophilized leaf samples were sent to Diversity Array Technology (DArT)®, Canberra, Australia, for DNA extraction, library construction, and SNP marker development.

The DNA was extracted using a technique developed by Intertek-AgriTech (http://www.intertek.com/agriculture/agritech/) and based on the LGC oKtopure^{TM} automated high-throughput 'sbeadex^{TM}' DNA extraction and purification system (https://www.biosearchtech.com/). Magnetic separation is used in the 'sbeadex^{TM}' technique to prepare nucleic acids. The first stage in this process is to homogenize leaf tissue samples in 96 deep-well plates using steel bead grinding. The ground tissue is treated with a DNA extraction buffer using LGC's 'sbeadex^{TM}' kit for plant DNA preparation (https://www.biosearchtech.com/). Finally, super-paramagnetic particles coated with 'sbeadexTM' surface chemistry that catches nucleic acids from a sample are used to purify extracted DNA. Purified DNA is eluted and used in downstream procedures.

High-throughput genotyping was conducted in 96 plex DArTseq protocol, and SNPs were called using the DArT's proprietary software, DArTSoft, as described by Killian et al. [46]. Each sequencing result's reads and tags were aligned to the soybean reference genome [59].

SNP Marker Quality Control

Single-row format data from DArT were initially converted into HapMap and variant call format (VCF) formats using KDcompute (https://kdcompute.seqart.net/kdcompute, accessed on 07/06/2024). SNP-derived markers were then first filtered using PLINK 1.9 and VCFtools, on the basis of the call rate of the raw data [60]. The SNP markers with call rates ranging from 0.80 to 1.0 were selected for further quality control analyses. Duplicate SNP markers across the chromosomes were removed. This process involved removing markers with

minor allele frequencies of less than 5%, markers and genotypes with more than 20% missing data, and those with a low coverage read depth of less than 5 [61, 62].

Statistical Analyses

The structure and pattern of genetic diversity within soybean genotypes were assessed via genotypic data generated on the basis of SNP markers. VCFtools and PLINK 1.9 were used to estimate summary statistics such as observed and expected heterozygosity, minor allele frequency (MAF), and polymorphic information content (PIC). The genotypic data was formatted in dosage format (0,1,2) using the recodeA function in Plink, where 0 is the homozygote reference, 1 is the homozygote alternative, and 2 is the heterozygote. The generated dosage format was then analyzed with the vegan library in R to estimate several genetic diversity indices, including the Shannon-Wiener index (H′), the inverse Simpson index (1/D) and the Alpha diversity index (A). These indices were used to assess the soybean genotypes' overall genetic diversity and allelic richness, following the methodology outlined by Oksanen et al. [63]. The SNP distribution and density plot of the SNP markers across the 20 chromosomes of the soybean genome was constructed via the CMplot package [64]. The SNP markers data were subjected to population structure analysis following the method described by Agre et al. [65]. By testing cluster numbers ranging from 2 to 10, the optimal number of clusters was identified through k-means analysis, employing cross-validation on the basis of the Bayesian information criterion (BIC). Each soybean genotype was assigned to its respective cluster if it had at least 70% ancestry probability. Genotypes with less than 70% ancestry were considered as admixed. The diversity pattern revealed through population structure analysis was further supported by discriminant analysis of the principal component (DAPC) via the Adegenet package in R [66]. DAPC, which uses the k-means clustering method, aims to minimize variance within clusters while maximizing variance between clusters [67]. Pairwise genetic dissimilarity distances (identity-by-state, IBS) were calculated via the

Jaccard method, implemented in the Philentropy R package [68]. A Ward's minimum variance hierarchical cluster dendrogram was then constructed from the Jaccard dissimilarity matrix using the Analyses of Phylogenetics and Evolution (APE) package in R [69]. Principal component analysis (PCA) was subsequently conducted to determine the genetic relationships among 147 soybean genotypes via the FactoMineR [70] and FactoExtra R packages [71]. Molecular variance analysis (AMOVA) and calculation of the coefficient of genetic differentiation among populations (PhiPT) were performed to investigate the distribution of genetic diversity among and within hierarchical populations via GenAlEx software (v.6.51) [72].

Results

Genetic diversity indices

A total of 59,126 SNP markers from the 147 soybean genotypes were originally generated via the Diversity Arrays Technology (DArT) platform. The transformation of these allelic sequences into genotypic data resulted in a raw data file of 53,418 SNPs, and after quality control analysis (SNP filtering), only 7,083 SNP markers were retained for further analyses. These markers were unequally distributed across the 20 soybean chromosomes (Fig 1; Table 1). The genome-wide SNP density plot indicated that chromosome 18 had the highest concentration of SNPs, accounting for 7.6% of the total number of markers with 538 SNPs. In contrast, chromosome 12 had the lowest concentration, with only 3.17% of the SNPs, totaling 225 markers (Fig 1). The diversity indices for the SNP marker presented a polymorphic information content (PIC) value of 0.277, ranging from 0.262 to 0.293. The MAF averaged 0.254 across all the markers. The observed heterozygosity (Ho) ranged from 0.093 to 0.124, with an average value of 0.110. The expected heterozygosity (He) varied between 0.322 and 0.371, with an average of 0.344 (Table 1 and S1 Fig). The Shannon diversity index (H′) index ranged from 8.505 to 8.704, with a mean value of 8.597. The inverse Simpon's index (1/D) had

an average of 5349.145, with a range from 4903.021 to 5824.754. The Alpha diversity index (A) varied between 4218.816 and 7568.063, with a mean value of 4950.72.

Fig 1. Distribution and density of filtered SNPs across 20 soybean chromosomes. The horizontal axis represents the chromosome length, the SNP density in each region is indicated at the bottom right

Table 1. Summary statistics of diversity indices of 147 soybean accessions based on SNP markers.

Chr	Nbr of SNPs	Ho	He	MAF	PIC	H′	1/D	A
1	260	0.116	0.359	0.267	0.287	5.304	198.66	188.77
2	334	0.111	0.335	0.250	0.269	5.546	253.235	235.059
3	345	0.105	0.338	0.246	0.272	5.579	262.490	242.013
4	328	0.094	0.322	0.231	0.262	5.555	256.267	231.879
5	285	0.104	0.349	0.256	0.281	5.382	215.95	198.54
6	462	0.095	0.34	0.251	0.274	5.886	356.442	325.612
7	296	0.093	0.323	0.230	0.263	5.438	228.309	205.593
8	440	0.108	0.334	0.241	0.270	5.838	339.115	313.574
9	374	0.108	0.371	0.288	0.293	5.619	271.903	256.762
10	247	0.108	0.348	0.259	0.278	5.225	184.079	170.973
11	274	0.105	0.339	0.250	0.272	5.340	206.522	189.444
12	225	0.113	0.354	0.265	0.284	5.139	168.733	157.169
13	470	0.124	0.357	0.273	0.284	5.870	349.287	332.682
14	399	0.112	0.357	0.265	0.287	5.712	299.103	332.682
15	435	0.112	0.345	0.249	0.278	5.820	333.216	310.035
16	463	0.119	0.333	0.238	0.270	5.876	352.129	326.264
17	290	0.124	0.357	0.269	0.284	5.405	220.020	207.293
18	538	0.114	0.344	0.255	0.277	6.002	399.211	37098
19	310	0.112	0.353	0.232	0.284	5.418	223.069	208.533
20	308	0.111	0.336	0.250	0.271	5.460	232.811	216.033
**Min**	225	0.094	0.322	0.230	0.262	8.505	4903.021	4218.816
**Mean**	354.15	0.110	0.344	0.254	0.277	8.597	5349.145	4950.728
**Max**	538	0.124	0.371	0.228	0.293	8.704	5824.754	7568.063

Chr: chromosome, Nbr of SNPs: Number of single nucleotide polymorphism, Ho: observed heterozygosity; He: expected heterozygosity; MAF: minor allelic frequency; PIC: polymorphism information content, H′: Shannon-Wiener index, 1/D: inverse Simpson index, A: Alpha diversity index, Max= maximum, Min= minimum

Population structure of 147 soybean breeding lines

Various complementary methods, including a model-based Bayesian approach in ADMIXTURE, DAPC, and PCA), were utilized to analyze the population structure of the 147 soybean accessions. Based on the admixture results, four subpopulations (K=4) were identified (Fig 2). Similarly, DAPC revealed four genetic groups (Fig 3), following a sharp decline in the Bayesian information criterion (BIC) versus the number of cluster plots (S2 Fig). There was a disparity in how the soybean accessions were assigned to the identified genetic groups between the ADMIXTURE and DAPC results. This disparity may be related to the DAPC analysis, which assigned the 147 soybean genotypes into distinct groups. In contrast, ADMIXTURE assigned 57% of the accessions (84 genotypes) to the four subpopulations on the basis of a membership probability of 70%, whereas the remaining 43% (63 lines) of the collection were classed as admixtures.

Fig 2. Population structure of 147 soybean breeding lines from the IITA breeding program, Ibadan on the basis of ADMIXTURE analysis with the subpopulations set at K = 4 via 7,083 high-quality SNPs. The colors correspond to the four subpopulations: Subpopulation 1 (red), Subpopulation 2 (blue), Subpopulation 3 (green) and Subpopulation 4 (cyan), determined by -a membership coefficient greater than70%.

Fig 3. Summary of discriminant analysis of principal component (DAPC) for 147 soybean accessions, illustrating the ordination plot of DAPC for the four groups. Eigenvalues are displayed in the upper-left inset. Genetic groups or clusters are represented by distinct colors and inertia ellipses, with individual genotypes indicated by dots.

Hierarchical cluster (HC) analysis grouped all 147 soybean genotypes into three major genetic groups or clusters (Fig 4 and S3 Fig). Cluster 1 contained 87 genotypes, predominantly IITA breeding lines, except a single genotype, ‘SONGDA’ introduced from Ghana, which was originally an IITA breeding line sent to Ghana in variety trials. The 86 IITA genotypes were mainly TGx (Tropical *Glycine max*) varieties or progenies resulting from crosses between two TGx parental lines (S1 Table). The HC analysis grouped these 87 genotypes into Cluster 1, while the DAPC divided them into two distinct clusters, represented as Clusters 3 and 4 (Figure

3). According to the ADMIXTURE analysis, 36 of the 87 genotypes in Cluster 1, including the unique Ghana genotype, were classified as admixtures. The remaining 51 accessions were assigned to the blue and cyan groups, with 34 and 17 genotypes, respectively (Figure 2). Cluster 2 comprised 34 accessions, including 16 IITA-breeding lines, 16 of the 17 genotypes sourced from the USDA soybean genetic resource center, and one variety Sc-Signa from SeedCo (a private Company) and MAKSOY-4N from Makere University, Uganda. The 16 IITA breeding lines consisted of progenies derived from various parental lines, including TGx, ZIGx, SPSOY, CIMARRONA, PI567090, SOYICA and ST SUPREMA (S1 Table). Among the 34 genotypes in Cluster 2 identified by HC analysis, 15, exclusively IITA breeding lines, were clustered by ADMIXTURE analysis in subpopulation 1 (red) (Figure 2). The remaining 19 genotypes, which included one IITA breeding line, 16 from Columbia, and the unique genotypes from SeedCo and Uganda, were assigned as admixes by ADMIXTURE analysis. On the other hand, the DAPC analysis placed all Cluster 2 genotypes into Cluster 1 (Figure 3). The 26 genotypes assigned to Cluster 3 by HC analysis included 25 IITA breeding lines and one Columbia genotype (Panaroma-3). The IITA breeding lines were a mix of pure TGx parents and backcross progenies, derived from crosses between TGx lines and other parental lines, such as ST SUPREMA, CIMARONA, SOYICA, ZIGx, LG-12, and AS-G (S1 Table). The DAPC analysis classified these 26 genotypes from Cluster 3 in the HC analysis into Cluster 2 (Fig 3). Moreover, the ADMIXTURE analysis placed 18 of them into a specific group (green) (Fig 3), whereas the remaining 8, including the unique USDA genotype (Panaroma-3), were categorized as admixtures.

Fig4. Hierarchical clustering analysis based on 7,083 DArT-SNP markers, depicting the genetic relationships among 147 soybean accessions from the IITA, Ibadan breeding programme.

Principal component analysis (PCA) revealed that the first and second components (PC1 and PC2) accounted for 45.2% and 15.9% of the total molecular variation, respectively, together explaining 61.1% of the overall observed variation (Figure 5). Although all the

genotypes within each cluster were grouped, they exhibited some heterogeneity. The genotypes classified as admixtures were identified as admixed groups in the PCA (Figure 5).

Fig 5. Principal component analysis plot showing the clustering of 147 soybean breeding accessions into four clusters. Each cluster is represented by a distinct color: Cluster 1 (red), Cluster 2 (yellow), Cluster 3 (green), Cluster 4 (blue), and admixed individuals (pink).

Genetic distance and differentiation of soybean accessions

A pairwise dissimilarity genetic distance matrix revealed that the genetic distance among the 147 soybean genotypes ranged from 0.012 to 0.452, with an average distance of 0.333. The greatest genetic distance of 0.452 was found between the USDA genotype TGx 2029-39F (Cluster 2) and two IITA breeding lines, TGx 2002-89 GN and TGx1988-5FxTGx1989-19F-9, both in Cluster 1. In contrast, the lowest genetic distance (0.012) was observed between two IITA lines, TGx 2002-89 GN and TGx 2002-90 GN, both of which belonged to cluster 1. Within Cluster 1, the genetic distances ranged from 0.012 to 0.452 with an average of 0.337. Cluster 2 presented genetic distances ranging from 0.015 to 0.452, with an average of 0.367. For Cluster 3, the distances ranged from 0.017 to 0.433, with an average of 0.355.

The analysis of molecular variance (AMOVA) revealed that 77% of the total genetic variability was partitioned as within-population variation, which was significantly greater than the 23% partitioned among among-populations variation (Table 2). The overall genetic differentiation (PhiPT) and gene flow (Nm) for the 147 soybean genotypes were 0.233 ($p < 0.001$) and 1.649, respectively.

Table 2. Analysis of molecular variance (AMOVA), population differentiation (PhiPT), and gene flow (Nm) within and among soybean populations

Source of Variation		DF	SS	MS	Est. Var.	%
Within populations		144	184936.218	1284.279	1284.279	77

Among populations		2	34906.864	17453.432	389.395	23
Total		146	219843.082		1673.674	100
Statistical test	PhiPT				0.233	
	p (rand >= data)				0.001	
	Nm (haploid)				1.649	

DF, degrees of freedom; SS, sum of squares; MS, mean square deviation; Est.Var., estimated variance component; percentage of the total variance (%) contributed by each component and significance of variance (p-value); Nm, gene flow.

The pairwise population differentiation (PhiPT) estimates revealed that the highest degree of differentiation (0.267) was observed between populations 1 and 3, whereas the lowest degree of differentiation (0.200) occurred between populations 1 and 2. The genetic differentiation between population 2 and population 3 was 0.244. On the other hand, the pairwise population estimates of gene flow (Nm) for the three populations ranged from 1.376 to 1.998 migrants per population (Table 3).

Table 3: Pairwise population comparison using population differentiation (PhiPT), and gene flow (Nm) values based on 999 permutations from AMOVA according to Hierarchical Cluster analysis

Population 1	Population 2	PhiPT	Nm
Cluster 1	Cluster 2	0.200***	1.998
Cluster 1	Cluster 3	0.267***	1.376
Cluster 2	Cluster 3	0.244***	1.546

***; PhiPT values significantly greater than 0, $p < 0.001$

Discussion

Studying the genetic diversity of germplasm or breeding material is the best approach for understanding the existing genetic variation and effectively managing genetic resources to enhance breeding programs [73, 74]. Hence, plant breeders need such genetic analysis to execute strategic target selection and integration while maintaining significant economic traits associated with distinct crops [75].

The average value of 0.277 indicates that the markers used in this study were both informative and polymorphic. Given the bi-allelic nature of SNPs, where the PIC cannot exceed 0.5 [76], the PIC values observed in this study are suitable for differentiating the 147 soybean

accessions. Similar results have been reported in soybean studies, including Abebe et al. [2], who found a mean PIC value of 0.25 for elite soybean lines developed by IITA, and Lee et al [77], who reported a PIC value of 0.22 when evaluating 228 soybean genotypes. Tsindi et al.[78] also reported a PIC value of 0.2 for 210 South African soybean genotypes. In other self-pollinated crops, Singh et al. [76] reported a mean PIC value of 0.23 in rice. This study also demonstrated the possibility of using the selected DArTseq-SNP markers for genomic investigations in soybeans, which may serve as a foundation for future breeding efforts in the IITA soybean breeding program and conservation initiatives in Nigeria. The MAF value measures the selective ability of the marker. Owing to the bi-allelic nature of SNP markers, the MAF closest to 0.5, is best. The high average MAF value of 0.254 observed in this study indicates valuable genes can be exploited from those genotypes [75]. Compared to the results based on SNP markers reported by Hao et al. [79], our MAF values are lower. Their study revealed that the MAF ranged from 0.102 to 0.50 in soybean landraces, with an average value of 0.291. This difference might be because the materials used in the present study were advanced breeding lines, whereas Hao et al. [79] focused mainly on landraces. The average expected heterozygosity (He) of 0.344 indicates high genetic diversity within the soybean accessions, which can be effectively used for soybean improvement [75]. The Shannon-Wiever diversity index (H′), which quantifies the entropy or uncertainty in the genetic composition of a population, has a mean value of 8.597. This relatively high value indicates a genetically diverse population with various alleles and genotypes. Such a result suggests that our soybean genotypes are built on a genetic foundation, with a broad range of genetic types contributing to its overall diversity. Similarly, the inverse Simpson index (1/D), with its high mean value of 5349.145, reinforces the genetic diversity within our soybean population. It also implies a balanced distribution of genotypes without any single genotype dominating, reflecting a well-represented mix of genetic types. Comparable findings have been reported in studies on soybean [80, 81], maize [82], rice [83], and wheat [84]. Furthermore, the alpha diversity index (A), which assesses both the richness (the number of distinct genotypes or alleles) and evenness (the relative abundance of each genotype/allele) within a population, also exhibits a high mean value (4950.720). This suggests that while genetic diversity may vary across different subpopulations or sites, the overall population remains genetically rich, as reported by Adejumobi et al [85].

Analyzing population structure via SNP markers offers helpful information for preserving and tracking the genetic diversity essential for an effective breeding program [86]. ADMIXTURE

and DAPC analyses were used to determine the population structure, revealing the presence of four major populations (K = 4) for the 147 soybean genotypes. However, previous studies [2] and [6] reported different ADMIXTURES results, with ΔK values of 3 and 6, respectively. Considerable levels of admixture (42.87%) were detected among the genotypes, which likely resulted from historical gene flow, breeding practices, and inherent genetic diversity within and between the soybean populations [57]. Chander et al. [6] reported similar levels of admixture in their study of 165 soybean genotypes, which primarily consisted of IITA-bred soybean varieties. In contrast to the results of the ADMIXTURE and DAPC analyses, the hierarchical cluster (HC) method classified the 147 genotypes into three major clusters, suggesting that this could represent the optimal number of genetic clusters within the soybean accessions studied [78]. These results highlight the effectiveness of SNP markers in identifying superior accessions that have the potential to enhance the genetic diversity of the soybean population [87]. Furthermore, the results emphasize the importance of the distinct pedigrees of the soybean genotypes in maintaining genetic variation, as genotypes with similar pedigrees tended to cluster together based on their SNP profiles. Similar clustering patterns, which reflect the genetic origin of the accessions, have been reported in soybean [79–88-89,] as well as in other legume species such as cowpea [90, 91] and sesame [92]. Grouping the 147 genotypes into four distinct clusters within the first two principal components accounting for more than 60% of the total genetic variation indicates a high level of variation among the genotypes across the clusters, but high relatedness within a specific cluster. These results suggest that the genotypes within a given cluster share significant genetic similarities, making them potentially valuable for enhancing the genetic diversity of soybean breeding programs through hybridization. In support of this, Bakayoko et al. [60] highlighted that genotypes within the same cluster are genetically similar and thus could play a crucial role in genetic improvement efforts.

The results from the analysis of molecular variance (AMOVA) suggest a significant level of gene flow, with 23% of the total variation attributable to differences among populations, while 77% of the variation was observed within populations. This indicates that the majority of genetic variation resides within populations, but there is still considerable variation between them, supporting the presence of gene flow. These results are further corroborated by the gene flow (Nm) value, which plays a key role in enhancing the genetic diversity and influencing genetic differentiation of plant populations and is a crucial factor influencing genetic differentiation [93]. When Nm is greater than 1, gene flow is sufficient to counteract the effects

of genetic drift. In this study, the average gene flow value (Nm = 1.649) suggests that the soybean populations are not yet significantly impacted by genetic drift. Similar findings have been reported in previous studies on soybean [9- 89– 94,95] and other crops, such as Camelina sativa [96], wheat [97], rice [14], cowpea [74– 98], and potatoes [99]. The PhiPT value (an analogue of the fixation index Fst) of 0.23 indicates a high level of genetic differentiation among populations, suggesting limited gene exchange. In general, low Fst values close to 0 suggest that subpopulations are genetically similar, with minimal divergence, whereas an Fst of 1 indicates complete genetic fixation within subpopulations [92– 100]. This analysis also revealed significant genetic differentiation between populations 1 and 2 and between populations 2 and 3. Moreover, the differentiation between populations 1 and 3 was particularly pronounced. This substantial genetic diversity among all pairwise populations highlights the considerable diversity within the soybean accession and emphasizes the effectiveness of the selected markers for future research on soybean genetic diversity [2]. Consequently, hybridizing genotypes from different populations could introduce valuable genetic variation, enhancing genetic gain through focused selection [9].

Conclusion

The soybean lines analyzed in this study exhibit high levels of polymorphism and genetic diversity, reflecting considerable genetic variability within the population. A distinct genetic structure was observed among the sub-populations, which were grouped based on their pedigree or geographic origin. The distribution of soybean genotypes across major clusters or sub-populations, as revealed by multivariate analyses, highlights the success of IITA's plant breeding efforts in creating a diverse genetic base. This diversity has been achieved while maintaining a focus on enhancing local adaptation to various agroecological zones within soybean-growing areas of West Africa. The diverse nature of materials used in this study, suggests these materials serve as valuable sources of genetic variation. These genotypes potentially harbor contrasting parental traits and novel alleles relevant to economically significant characteristics such as yield, drought resistance, and pod shattering. These findings present an opportunity for soybean breeders to improve the efficient selection of parental lines.

Moreover, the study emphasizes the potential need to integrate exotic germplasm into breeding programs to further enrich the genetic diversity base of soybeans in the region.

Acknowledgements

We gratefully acknowledge the technical support the soybean breeding team provided in establishing the trials and leaf sample collection.

References

1. Shete R, Borale S, Andhale G, Girase V. Screening of soybean genotypes for pod-shattering tolerance and association of different traits with seed yield. The Pharma Innovation Journal. 2023; 12: 1548-1551.

2. Abebe AT, Kolawole AO, Unachukwu N, Chigeza G, Tefera H, Gedil M. Assessment of diversity in tropical soybean (*Glycine max* (L.) Merr.) varieties and elite breeding lines using single nucleotide polymorphism markers. Plant Genetic Resources: Characterisation and Utilisation. 2021; 19: 20-28. https://doi.org/10.1017/S1479262121000034

3. Dean F, Science H, Ranga ANG. Soyabean the Miracle Golden Bean in Indian Foods. Acta Scientific Nutritional Health. 2019; 3: 44-49.

4. Tolorunse KD, Joseph EC, Gana AS, Azuh VO. Molecular characterization of soybean (*Glycine max* (L.) Merril) genotypes using SSR markers. Nigeria Journal of Plant Breeding. 2022; 1: 12-17.

5. Andrijanić Z, Nazzicari N, Šarčević H, Sudarić A, Annicchiarico P, Pejić I. Genetic Diversity and Population Structure of European Soybean Germplasm Revealed by Single Nucleotide Polymorphism. Plants. 2023; 12: 1837. https://doi.org/10.3390/plants12091837

6. Chander S, Lu A, Gedil M, Shah T, Otusanya GO, Asiedu R, et al. Genetic Diversity and Population Structure of Soybean Lines Adapted to Sub-Saharan Africa Using Single Nucleotide (SNP) Markers. Agronomy. 2021; 11: 604. https://doi.org/10.3390/agronomy11030604

7. Ronner E, Frankea AC, Vanlauwec B, Dianda M, Edehe E, Ukeme B, et al. Understanding

Formatted: English (United Kingdom)

variability in soybean yield and response to P-fertilizer and rhizobium inoculants on farmers' fields in northern Nigeria. Field Crops Research. 2016; 186: 133-145. https://doi.org/10.1016/j.fcr.2015.10.023

8. Khojely DM, Ibrahim SE, Sapey E, Han T. History, current status, and prospects of soybean production and research in sub-Saharan Africa. The Crop Journal. 2018; 6: 226-235. https://doi.org/https://doi.org/10.1016/j.cj.2018.03.006

9. Shaibu AS, Ibrahim H, Miko ZL, Mohammed IB, Mohammed SG, Yusuf HL, et al. Assessment of the Genetic Structure and Diversity of Soybean and Single Nucleotide Polymorphism Markers. Plants. 2022; 11: 68. https:// doi.org/10.3390/plants11010068

10. Bhandari HR, Bhanu NA, Srivastava K, Singh MN, Shreya, Hemantaranjan A. Assessment of Genetic Diversity in Crop Plants - an overview. Adv Plants Agric Res. 2017; 7: 279-286. https://doi.org/10.15406/apar.2017.07.00255

11. Zatybekov A, Yermagambetova M, Genievskaya Y, Didorenko S, Growing P. Genetic Diversity Analysis of Soybean Collection Using Simple Sequence Repeat Markers. Plants. 2023; 12: 3445. https://doi.org/10.3390/plants12193445

12. Onda Y, Mochida K. Exploring Genetic Diversity in Plants Using High-Throughput Sequencing Techniques. Current Genomics. 2016; 17: 358–367. https://doi.org/10.2174/1389202917666160331202742

13. Schaal BA, Hayworth DA, Olsen KM, Rauscher JT, Smith WA. Phylogeographic Studies in Plants. Molecular Ecology. 1998; 7: 465–474.

14. Adeboye KA, Oyedeji OE, Alqudah AM, Börne A, Oduwaye O, Adebambo O, et al. Genetic structure and diversity of upland rice germplasm using diversity array technology (DArT)-based single nucleotide polymorphism (SNP) markers. Plant Genetic Resources: Characterisation and Utilisation. 2020; 18: 343–350. https://doi.org/10.1017/S1479262120000362

15. Liu Y, Lai Y, Wang C, Li X, Liu S, He C. Fine mapping and candidate gene analysis of qRT9, a novel major QTL with pleiotropism for yield-related traits in soybean. BMC Genomics. 2018; 19: 217.

16. Bressan EA, Neto TB, Zucchi MI, Rabello RJ, Veasey EA. Genetic structure and diversity in the *Dioscorea cayenensis*/*D. rotundata* complex revealed by morphological and

isozyme markers. Genet. Mol. Res., 2014, 13: 425–437. https://doi.org/10.4238/2014.January.21.10

17. Bressan EA, Neto TB, Zucchi MI, Rabello RJ, Veasey EA. Morphological variation and isozyme diversity in *Dioscorea alata* L. landraces from Vale do Ribeira, Brazil. Sci. Agric. 2011; 68: 494–502. https://doi.org/10.1590/S0103-90162011000400016
18. Zannou A, Agbicodo E, Zoundjihékpon J, Struik PC, Ahanchédé A, et al. Genetic variability in yam cultivars from the guinea- Sudan zone of Benin assessed by random amplified polymorphic DNA. Afr. J. Biotechnol. 2009; 8: 26–36. https://doi.org/10.5897/AJB2009.000-9005
19. Terauchi R, Chikaleke VA, Thottappilly G, Hahn SK. Origin and phylogeny of Guinea yams as revealed by RFLP analysis of chloroplast DNA and nuclear ribosomal DNA. Theor. Appl. Genet. 1992; 83: 743–751. https://doi.org/10.1007/BF00226693
20. Sonibare MA, Asiedu R, Albach DC. Genetic diversity of *Dioscorea dumetorum* (Kunth) Pax using Amplified Fragment Length Polymorphisms (AFLP) and cpDNA. Biochem. Syst. Ecol. 2010; 38: 320–334. https://doi.org/10.1016/j.bse.2010.01.012
21. Arnau G, Bhattacharjee R, Sheela MN, Chair H, Malapa R, Lebot V, et al. Understanding the genetic diversity and population structure of yam (*Dioscorea alata* L.) using microsatellite markers. PLoS ONE 2017; 12: 1–17. https://doi.org/10.1371/journal.pone.0174150
22. Mulualem T, Mekbib F, Shimelis H, Gebre E, Amelework B. Genetic diversity of yam (*Dioscorea* spp.) landrace collections from Ethiopia using simple sequence repeat markers. Aust. J. Crop Sci. 2018; 12: 1222–1230. https://doi.org/10.21475/ajcs.18.12.08.PNE885
23. Silva DM, Siqueira MVBM, Carrasco NF, Mantello CC, Nascimento WF, Veasey EA. Genetic diversity among air yam (*Dioscorea bulbifera*) varieties based on single sequence repeat markers. Genet. Mol. Res. 2016; 15: 1–12. https://doi.org/10.4238/gmr.15027929
24. Otto, E.; Anokye, M.; Asare, P.A.; Tetteh, J.P. Molecular Categorization of some Water Yam (*Dioscorea alata* L.) Germplasm in Ghana using Microsatellite (SSR) Markers. *J. Agric. Sci.* **2015**, 7(10).
25. Siqueira MV, Dequigiovanni G, Corazon-Guivin MA, Feltran JC, Veasey EA. DNA fingerprinting of water yam (*Dioscorea alata*) cultivars in Brazil based on microsatellite markers. Hortic. Bras. 2012; 30: 653–659. https://doi.org/10.1590/s0102-05362012000400015

Formatted: French (France)

26. Wang M, Li RZ, Yang WM, Du WJ. Assessing the genetic diversity of cultivars and wild soybeans using SSR markers. Afr J Biotechnol., 2010; 9:4857-4866.

27. Guan R, Chang R, Li Y, Wang L, Liu Z, Qiu L. Genetic diversity comparison between Chinese and Japanese soybeans (*Glycine max* (L.) Merr.) revealed by nuclear SSRs. Genet Resour Crop Evol., 2010; 57:229-242

28. Kujane K, Sedibe MM, Mofokeng A. Genetic diversity analysis of soybean (Glycine max (L.) Merr.) genotypes making use of SSR markers. Australian Journal of Crop Science, 2019; 13: 1113–1119. https://doi.org/10.21475/ajcs.19.13.07.p1638

29. Acinas SG, Sarma-Rupavtarm R, Klepac-Ceraj V, Polz MF. PCR-induced sequence artefacts and bias: Insights from comparison of two 16s rRNA clone libraries constructed from the same sample. Appl. Env. Microb. 2005; 71: 8966–8969. https://doi.org/10.1128/AEM.71.12.8966-8969.2005

30. Brakenhoff RH, Schoenmakers JGG, Lubsen NH. Chimeric cDNA clones: A novel PCR artefact. Nucleic Acids Res. 1991; 19: 1949. https://doi.org/10.1093/nar/19.8.1949

31. Cline J, Braman JC, Hogrefe HH. PCR fidelity of Pfu DNA polymerase and other thermostable DNA polymerases. Nucleic Acids Res. 1996; 24: 3546–3551. https://doi.org/10.1093/nar/24.18.3546

32. Kulibaba RA, Liashenko YV. Influence of the PCR artefacts on the genotyping efficiency by the microsatellite loci using native polyacrylamide gel electrophoresis. Cytol. Genet. 2016; 50: 162–167. https://doi.org/10.3103/S0095452716030087

33. Tsykun T, Rellstab C, Dutech C, Sipos G, Prospero S. Comparative assessment of SSR and SNP markers for inferring the population genetic structure of the common fungus *Armillaria cepistipes*. Heredity 2017; 119: 371–380. https://doi.org/10.1038/hdy.2017.48

34. Yu Z, Fredua-Agyema R, Hwang SF, Strelkov SE. Molecular genetic diversity and population structure analyses of rutabaga accessions from Nordic countries as revealed by single nucleotide polymorphism markers. BMC Genomics 2021; 22: 1–13. https://doi.org/10.1186/s12864-021-07762-4

35. Vivodík M, Balážová Ž, Chňapek M, Hromadová Z, Mikolášová L, Gálová Z. Molecular Characterization and Genetic Diversity Studies of Soybean (*Glycine max* L.) Cultivars Using Rapid Markers. Journal of Microbiology, Biotechnology and Food Sciences, 2022; 12: 1–5. https://doi.org/10.55251/jmbfs.9219

Formatted: English (United Kingdom)

36. Vignal A, Milan D, SanCristobal M, Eggen A. A review on SNP and other types of molecular markers and their use in animals. Genetics 2002; *34*: 275–305. https://doi.org/10.1051/gse

37. Verma S, Gupta S, Bandhiwal N, Kumar T, Bharadwaj C, Bhatia, S. High-density linkage map construction and mapping of seed trait QTLs in chickpea (*Cicer arietinum* L.) using genotyping-by-sequencing (GBS). Sci. Rep., 2015; 5: 17512.

38. Fischer MC, Rellstab C, Leuzinger M, Roumet M, Gugerli F, Shimizu KK, et al. Estimating genomic diversity and population differentiation - an empirical comparison of microsatellite and SNP variation in Arabidopsis halleri. BMC Genomics. 2017; 18: 1–15. https://doi.org/10.1186/s12864-016-3459-7

39. Lam H-M, Xu X, Liu X, Chen W, Yang G, Wong F-L, et al. Resequencing of 31 wild and cultivated soybean genomes identifies patterns of genetic diversity and selection. Nature Genetics. 2010; 42: 1053–1059. https://doi.org/10.1038/ng.715

40. Zhou Z, Jiang Y, Wang Z, Gou Z, Lyu J, Li W, et al. Resequencing 302 wild and cultivated accessions identifies genes related to domestication and improvement in soybean. Nature Biotechnology. 2015; 33: 408–414. https://doi.org/10.1038/nbt.3096

41. dos Santos JVM, Valliyodan B, Joshi T, Khan SM, Liu Y, Wang J, et al. Evaluation of genetic variation among Brazilian soybean cultivars through genome resequencing. BMC Genomics. 2016; 17: 1–18. https://doi.org/10.1186/s12864-016-2431-x

42. Valliyodan B, Brown AV, Wang J, Patil G, Liu Y, Otyama PI, et al. Genetic variation among 481 diverse soybean accessions, inferred from genomic re-sequencing. Scientific Data. 2021; .8: 1–9. https://doi.org/10.1038/s41597-021-00834-w

43. Torkamaneh D, Laroche J, Valliyodan B, O'Donoughue L, Cober E, Rajcan I, et al.. Soybean (Glycine max) Haplotype Map (GmHapMap): a universal resource for soybean translational and functional genomics. Plant Biotechnology Journal. 2021; 19: 324–334. https://doi.org/10.1111/pbi.13466

44. LeeY, Woo DU, Kang YJ. SoyDbean: a database for SNPs reconciliation by multiple versions of soybean reference genomes. Scientific Reports. 2023; 13: 1–10. https://doi.org/10.1038/s41598-023-42898-1

45. Yang X, Ren R, Ray R, Xu J, Li P, Zhang M, et al. Genetic diversity and population structure of core watermelon (*Citrullus lanatus*) genotypes using DArTseq-based SNPs.

Formatted: French (France)

Formatted: French (France)

Plant Genetic Resources: Characterisation and Utilisation. 2016; 14: 226–233. https://doi.org/10.1017/S1479262115000659

46. Kilian A, Wenzl P, Huttner E, Carling J, Xia L, Blois H, et al. Diversity arrays technology: a generic genome profiling technology on open platforms. Methods in Molecular Biology (Clifton, N.J.). 2012; 888: 67–89. https://doi.org/10.1007/978-1-61779-870-2_5

47. Chiemeke FK, Olasanmi B, Agre PA, Mushoriwa H, Chigeza G, Abebe AT. Genetic Diversity and Population Structure Analysis of Soybean [*Glycine max* (L.) Merrill] Genotypes Using Agro Morphological Traits and SNP Markers. Genes. 2024;15: 373. https://doi.org/10.3390/ genes15111373

48. Czembor E, Czembor JH, Suchecki R, Watson-Haigh NS. DArT-based evaluation of soybean germplasm from Polish Gene Bank. BMC Research Notes. 2021; 14: 1–6. https://doi.org/10.1186/s13104-021-05750-1

49. Tomkowiak A, Nowak B, Sobiech A, Bocianowski J, Wolko Ł, Spychała J. The Use of DArTseq Technology to Identify New SNP and SilicoDArT Markers Related to the Yield-Related Traits Components in Maize. Genes, 2022; 13: 848. https://doi.org/10.3390/genes13050848

50. Khodadadi M, Fotokian MH, Miransari M. Genetic diversity of wheat (*Triticum aestivum* L.) genotypes based on cluster and principal component analyses for breeding strategies. Aust. J. Crop Sci. 2011, 5, 17–24.

51. Sohail Q, Manickavelu A, Ban T. Genetic diversity analysis of Afghan wheat landraces (*Triticum aestivum*) using DArT markers. Genet. Resour. Crop Evol. 2015; 62: 1147–1157.

52. Adu BG, Adu Amoah R, Aboagye LM, Abdoul Aziz MG, Boampong R. High-Density DArT Markers and Phenotypic Characterization of Cowpea Accessions (*Vigna unguiculata* (L.) Walp). Advances in Agriculture.2021; ID 6336684, 12p. https://doi.org/10.1155/2021/6336684

53. Gbedevi KM, Boukar O, Ishikawa H, Abe A, Ongom PO. Genetic Diversity and Population Structure of Cowpea [*Vigna unguiculata* (L .) Walp .] Germplasm Collected from Togo Based on DArT Markers. Genes. 2021; 12: 1451.https://doi.org/10.3390/genes12091451

54. Mace ES, Xia L, Jordan DR, Halloran K, Parh DK, Huttner E, et al. DArT markers:

Diversity analyses and mapping in Sorghum bicolor. BMC Genom. 2008; 11: 1–11. doi:10.1186/1471-2164-9-26

55. Egea LA, Mérida-garcía R, Kilian A, Hernandez P, Dorado G. Assessment of Genetic Diversity and Structure of Large Garlic (*Allium sativum*) Germplasm Bank , by Diversity Arrays Technology Platform (DArTseq). Front. Genet. 2017; 8: 1–9. https://doi.org/10.3389/fgene.2017.00098

56. Alemu A, Feyissa T, Letta T, Abeyo B. Genetic diversity and population structure analysis based on the high-density SNP markers in Ethiopian durum wheat (*Triticum turgidum* ssp. durum). BMC Genet. 2020; 21:18. doi: 10.1186/s12863-020-0825-x.

57. Pritchard JK, Stephens M, Donnelly P. Inference of population structure using multilocus genotype data. Genetics. 2000; 155:945–959. https://doi.org/10.1093/genetics/155.2.945

58 Odong TL, Jansen J, Van Eeuwijk FA, van Hintum TJ. Quality of core collections for effective utilisation of genetic resources review, discussion and interpretation. Theoretical and Applied Genetics, 2013; 126: 289-305.

59. Schmutz J, Cannon SB, Schlueter J, Ma J, Mitros T, Nelson W, et al.. Genome sequence of the palaeopolyploid soybean. Nature. 2010; 463: 178–183. https://doi.org/10.1038/nature08670

60. Bakayoko L, N'Da Pokou D, Kouassi AB, Agre PA, Kouakou AM, Dibi KE, et al. Diversity Of Water Yam (*Dioscorea Alata* L.) Accessions from Côte D'ivoire Based On SNP Markers And Agronomic Traits. Plants. 2021; 10: 1–18. https://doi.org/10.3390/plants10122562

61. Danecek P, Auton A, Abecasis G, Albers CA, Banks E, DePristo MA, et al. The variant call format and VCFtools. Bioinformatics. 2011; 27: 2156–2158. https://doi.org/10.1093/bioinformatics/btr330

62. Purcell S, Neale B, Todd-Brown K, Thomas L, Ferreira MAR, Bender D, et al. PLINK: A tool set for whole-genome association and population-based linkage analyses. American Journal of Human Genetics. 2007; 81: 559–575. https://doi.org/10.1086/519795

63. Oksanen J; Guillaume BF, Friendly M, Roeland KP, Dan ML, Minchin PR, et al. Vegan: community ecology package 2019. R package version 2.5–5. Available at https://CRAN.R-project.org/package=vegan

64. Yin L. R package "CMPlots". 2019. Available: https://github.com/YinLiLin/RCMplot (Accessed June 2024).

65. Agre PA, Edemodu A, Obidiegwu JE, Adebola P, Asiedu R, Asfaw A. Variability and genetic merits of white Guinea yam landraces in Nigeria. Frontiers in Plant Science. 2023; 14: 1–12. https://doi.org/10.3389/fpls.2023.1051840

66. Salazar E, González M, Araya C, Mejía N, Carrasco B. Genetic diversity and intra-racial structure of Chilean Choclero corn (*Zea mays* L.) germplasm revealed by simple sequence repeat markers (SSRs). Scientia Horticulturae. 2017; 225: 620–629. https://doi.org/10.1016/j.scienta.2017.08.006

67. Jombart T. Adegenet: A R package for the multivariate analysis of genetic markers. Bioinformatics. 2008; 24: 1403–1405. https://doi.org/10.1093/bioinformatics/btn129

68. Drost HG. Philentropy: Information Theory and Distance Quantification with R. Journal of Open Source Software. 2018; 3: 765. https://doi.org/10.21105/joss.00765

69. Paradis E, Claude J, Strimmer K. APE : Analyses of Phylogenetics and Evolution in R language. Bioinformatics. 2004; 20: 289–290. https://doi.org/10.1093/bioinformatics/btg412

70. Lê S, Josse J, Husson F. FactoMineR: An R Package for Multivariate Analysis. Journal of Statistical Software. 2008; 25: 1–18.

71. Kassambara A, Mundt F. Factoextra: Extract and Visualize the Results of Multivariate Data Analyses. R Package Version 1.0.7. 2020. https://CRAN.R-project.org/package=factoextra

72. Peakall R, Smouse PE. GenALEx 6.5: Genetic analysis in Excel. Population genetic software for teaching and research —an update. Bioinformatics. 2012; 28: 2537–2539. https://doi.org/10.1093/bioinformatics/bts460

73. Al-Abdallat AM, Karadsheh A, Hadadd NI, Akash MW, Ceccarelli S, Baum M, et al. Assessment of genetic diversity and yield performance in Jordanian barley (*Hordeum vulgare* L.) landraces grown under Rainfed conditions. BMC Plant Biology. 2017; 17: 1–13. https://doi.org/10.1186/s12870-017-1140-1

74. Ketema S, Tesfaye B, Keneni G, Fenta BA, Assefa E, Greliche N, et al. DArTSeq SNP-based markers revealed high genetic diversity and structured population in Ethiopian

cowpea [*Vigna unguiculata* (L.) Walp] germplasms. PLoS ONE. 2020; 15: 1–20. https://doi.org/10.1371/journal.pone.0239122

75. Yirgu M, Kebede M, Feyissa T, Lakew B, Woldeyohannes AB, Fikere M. Single nucleotide polymorphism (SNP) markers for genetic diversity and population structure study in Ethiopian barley (*Hordeum vulgare* L.) germplasm. BMC Genomic Data. 2023; 24: 1–13. https://doi.org/10.1186/s12863-023-01109-6

76. Singh N, Choudhury DR, Singh AK, Kumar S, Srinivasan K, Tyagi RK, Singh NK, Singh R. Comparison of SSR and SNP markers in estimation of genetic diversity and population structure of Indian rice varieties. PLoS ONE. 2013; 8:1–14

77. Lee YG, Jeong N, Kim JH, Lee K, Kim KH, Pirani A, et al. Development, validation and genetic analysis of a large soybean SNP genotyping array. Plant Journal. 2015;. 81: 625–636. https://doi.org/10.1111/tpj.12755

78. Tsindi A, Eleblu JSY, Gasura E, Mushoriwa H, Tongoona P, Danquah EY, et al. Analysis of population structure and genetic diversity in a Southern African soybean collection based on single nucleotide polymorphism markers. CABI Agriculture and Bioscience. 2023; 4: 1–14. https://doi.org/10.1186/s43170-023-00158-2

79. Hao D, Cheng H, Yin Z, Cui S, Zhang D, Wang H, et al. Identification of single nucleotide polymorphisms and haplotypes associated with yield and yield components in soybean (*Glycine max*) landraces across multiple environments. Theoretical and Applied Genetics. 2012; 124: 447–458. DOI 10.1007/s00122-011-1719-0

80. Liu S, Zhang L, Zhao X. Genetic Diversity of Soybean Germplasm in China and its Implications for Breeding Programs. Journal of Agricultural Science. 2017; 155: 279–291.

81. Zhang, X, Zhao Y, Li F. Genetic Diversity and Population Structure of Soybean Genotypes under Different Ecological Zones in China. Plant Breeding. 2020; 139: 664–672.

82. Li W, Liu J, Yang Y. Genetic Diversity of Maize Populations in Northern China: Insights from SSR Markers. Crop Science.2019; .59: 1120–1130.

83. Singh R, Kumar R, Sharma R. Genetic Diversity of Rice (*Oryza sativa*) Germplasm and its Relationship with Agronomic Traits. Field Crops Research, 2020;. 253, 107790.

84. Wang Y, Zhou X, Zhao L. Genetic Diversity of Wheat (Triticum aestivum) Revealed by

Microsatellite Markers and Its Implications for Breeding Programs. PLOS ONE. 2018;. 13: e0207550.

85. Adejumobi II, Agre PA, Onautshu DO, Adheka, JG. Diversity , trait preferences , management and utilization of yams landraces (*Dioscorea species*): an orphan crop in DR Congo. Scientific Reports.2022; 12: 1–16. https://doi.org/10.1038/s41598-022-06265-w

86. Amponsah AE, Esuma W, Alicai T, Bhattacharjee R, Dramadri IO, Edema R, et al. Genetic diversity and population structure of Uganda's yam (*Dioscorea* spp.) genetic resource based on DArTseq. PLoS ONE. 2023; 18: 1–16. https://doi.org/10.1371/journal.pone.027753739

87. Adewumi AS, Adejumobi II, Opoku VA, Asare PA, Adu MO., Taah KJ, et al.. Exploring quantitative trait nucleotides associated with response to yam mosaic virus severity and tuber yield traits in *Dioscorea praehensilis* Benth . association scanning. Front. Hortic. 2024; 3: 1–15. https://doi.org/10.3389/fhort.2024.1459476

88. Lee G-A, Choi Y-M, Yi J-Y, Chung J-W, Lee M-C, Ma K-H, et al. Genetic diversity and population structure of Korean soybean collection Using 75 microsatellite markers. Korean J Crop Sci. 2014; 59: 492–497. https:// doi. org/ 10. 7740/ kjcs.

89. Liu Z, Li H, Wen Z, Fan X, Li Y, Guan R, Guo Y, et al. Comparison of genetic diversity between Chinese and American soybean (*Glycine max* (L.)) accession revealed by high-density SNPs. Front. Plant Sci. 2017. https:/ doi.org/ 10.3389/fpls.2017.02014.

90. Fatokun C, Girma G, Abberton M, Gedil M, Unachukwu N, Oyatomi O, et al. Genetic diversity and population structure of a mini-core subset from the world cowpea (*Vigna unguiculata* (L.) Walp.) germplasm collection. Sci Rep 2018; 8:1–10. https:// doi. org/ 10. 1038/ s41598- 018- 34555-9.

91. Sodedji FAK, Agbahoungba S, Agoyi EE, Kafoutchoni MK, Choi J, Nguetta SPA, Diversity, population structure, and linkage disequilibrium among cowpea accessions. Plant Genome. 2021. https:// doi. org/ 10. 1002/ tpg2. 20113.

92. Basak M, Uzun B, Yol E. Genetic diversity and population structure of the Mediterranean sesame core collection with use of genome-wide SNPs developed by double digest RAD Seq. PLoS ONE. 2019;14:1–15. https:// doi. org/ 10. 1371/ journal. pone. 02237 57.

Formatted: French (France)

Formatted: French (France)

93. Weising K, Nybom H, Wolff K, Kahl G. DNA Fingerprinting in Plants. CRC Press: Boca Raton, FL, USA; 2005: ISBN 978-0-8493-1488-9

94. Jeong SC, Moon JK, Park SK, Kim MS, Lee K, Lee SR, et al. Genetic diversity patterns and domestication origin of soybean. Theoretical and Applied Genetics. 2019; 132: 1179–1193. https://doi.org/10.1007/s00122-018-3271-7

95. Lukanda MM, Dramadri OI, Amponsah AE, Arusei P, Gitonga WH; Wasswa P, et al. Genetic diversity and population structure of Ugandan soybean (*Glycine max* (L.) germplasm based on DArTseq. Plant Molecular Biology Reporter. 2023; 41: 417-426. https://doi.org/10.1007/s11105-023-01375–9c

96. Luo Z, Brock J, Dyer JM, Kutchan T, Schachtman D, Augustin M, et al. Genetic diversity and population structure of a *Camelina sativa* spring panel. Frontiers in Plant Science. 2019; 10: 1–12. https://doi.org/10.3389/fpls.2019.00184

97. Eltaher S, Sallam A, Belamkar V, Emara HA, Nower AA, Salem KF, et al. Genetic diversity and population structure of F3:6 Nebraska Winter wheat genotypes using genotyping-by-sequencing. Frontiers in Genetics. 2018; 9: 1–9. https://doi.org/10.3389/fgene.2018.00076

98. Gomes AMF, Draper D, Talhinhas P, Batista Santos P, Simões F, Nhantumbo N, et al. Genetic diversity among cowpea (*Vigna unguiculata* (L.) Walp.) landraces suggests central Mozambique as an important hotspot of variation. Agronomy. 2020; 10: 1893. https://doi.org/10.3390/agronomy10121893

99. Lee KJ, Sebastin R, Cho GT, Yoon M, Lee GA, Hyun DY. Genetic Diversity and Population Structure of Potato Germplasm in RDA-Genebank: Utilization for Breeding and Conservation. Plants. 2021; 10: 752. https://doi.org/10.3390/ plants10040752

100. Mohammadi SA, Prasanna BM. Analysis of genetic diversity in crop plants— salient statistical tools and considerations. Crop Sci. 2003; 43:1235–48. https:// doi. org/ 10. 2135/ crops ci2003. 1235.

Formatted: English (United States)

Supporting information:

S1 Table. List of the soybean (*Glycine max* (L.) Merril) accessions used in this study and their respective origins. (DOCX)

S1 Fig. Summary statistics of 7,083 single nucleotide polymorphism (SNP) markers used for genotyping 147 soybean accessions. (a) Expected heterozygosity, (b) observed heterozygosity, (c) minor allele frequency and (d) polymorphic information content. (JPG)

S2 Fig. Graph showing the best k value via Bayesian information criterion analysis. (JPG)

S3 Fig. Silhouette graph showing the optimal number of hierarchical clusters. (JPG)

Author contributions:

Conceptualization: Tenena Silue, Paterne Angelot Agre, Bunmi Olasanmi, Abush Tesfaye Abebe.

Data curation: Tenena Silue.

Formal analysis: Tenena Silue, Adeyinka Saburi Adewumi, Paterne Angelot Agre.

Investigation: Tenena Silue

Methodology: Tenena Silue, Paterne Angelot Agre, Adeyinka Saburi Adewumi, Abush Tesfaye Abebe.

Resources: Abush Tesfaye Abebe

Software: Tenena Silue, Adeyinka Saburi Adewumi, Paterne Angelot Agre.

Supervision: Paterne Angelot Agre, Bunmi Olasanmi, Abush Tesfaye Abebe.

Validation: Tenena Silue, Adeyinka Adewumi, Paterne Angelot Agre, Abush Tesfaye Abebe.

Visualization: Tenena Silue, Adeyinka Adewumi, Paterne Angelot Agre.

Writing – original draft: Tenena Silue

Writing – review & editing: Tenena Silue, Adeyinka Saburi Adewumi, Idris Ishola Adejumobi, Paterne Angelot Agre, Bunmi Olasanmi, Abush Tesfaye Abebe.

Genetic diversity and population structure of soybean (*Glycine max* (L.) Merril) germplasm

Tenena Silue[1,2,*], Paterne Angelot Agre[2], Bunmi Olasanmi[3], Adeyinka Saburi Adewumi[2], Idris Ishola Adejumobi[2] and Abush Tesfaye Abebe [2,*]

Authors' Responses to Reviewers' comments

Reviewer #1: Dear …

The researchers analyzed the genetic diversity in soybean accessions using DArt technology. There isn't much new information available on the subject. The following adjustments are needed:

Abstract

1. The aim and the methods should be joined into a single phrase

Response: Thanks for your valuable observation. The aim and the methods have been joined into a single phrase. (Reference: Lines 22-25).

2. It would be helpful to include a brief conclusion about the obtained results

Response: This has been adequately taken care off (Lines 33-35).

Introduction

1. This section is too long and should be summarized

Response: Thank you for your observation. This section has been reduced to provide the most relevant information.

2. It is suggested that you use the most current references that you can find.

Response: Thank you for this comment, we have included as many recent references that are relevant to our work as possible while retaining the old references that are highly important in the revised version of the manuscript.

3. The authors should describe the DNA molecular marker related to this study

Response: Thank you for your observation, the requested information has been provided. (Lines 84-97) in the revised manuscript.

4. Detailed information regarding methodologies for genetic diversity should be provided.

Response: Thank you for your observation. The methodologies for assessing genetic diversity in crops were documented in lines 68 – 83.

5. The authors should provide the gap and hypothesis of this study.

Response: Thank you for your comment. The requested information has been updated. (Lines 98-104).

6. The authors should expressly indicate in a single sentence the originality of the work

Response: Thank you for the comments. This aspect has been addressed. (Lines 104-106).

7. What distinguishes this study is that the authors identified new tasks or linkages related to their investigation.

Response: We appreciate your comments. We have included the statement showing how our work was unique following the identified gap in the IITA soybean breeding program.

8. Both general and specific goals should be documented

Response: Thank you for your comment. This observation has been provided (Lines 109-110).

Material and methods

9. The tissue type used for DNA extraction should be described

Response: Thank you for your comment. The description of the tissue type used for DNA extraction was documented in lines 123-128.

10. The name of the commercial kit should be included

Response: Thank you for your comment. We have included the names of the commercial kits as requested in the revised manuscript. (Lines 129-141).

11. It is essential to cite your sources or references for each approach

Response: Thank you for your observation. Appropriate citations following the format of the PLOSONE journal have been made for the sources and approaches we explored and documented in the revised version of the manuscript.

12. The manufacture of all instruments and materials should be stated

Response: Thank you for your observation. We have tried our best to include the manufacturer of the instruments and materials that we could find as requested in the revised version of the

manuscript. Our limitation was that the genotyping procedure (DNA extraction and genomic scan) was all performed by the DArT laboratory.

13. Why did not the authors perform all measurements of genetic diversity indices like Na, Ne, and I?

Response: we appreciate your contribution in ensuring our manuscript is better improved. Our understanding of Na, Ne, and I points to population size, effective sample size, and inbreeding depression, respectively which we do not believe may provide newer and different information than the diversity parameters (allele frequency, PIC, H_O, H_e, 1/D, H', and A) we have explored in the study.

14. The authors should perform the computation of the genetic diversity index for population

Response: Thank you so much for this suggestion. We have included more population diversity assessment indices including the Shannon-Wiever index, inverse Simpson index, and Alpha diversity index to verify the results obtained from the marker diversity indices.

Results and discussion

15. This section is simply written and should be detailed

Response: Thanks so much for your observation. We have included more interpretations and tailored our discussion to the interpreted results in the revised version of the manuscript.

16. The results of the tables should be detailed

Response: Thanks so much for your suggestion. The results in the tables have been improved to include a better caption and footnote adjustment in the revised version of the manuscript as requested.

17. All abbreviations should be written in full name

Response: Thank you for your comment. Abbreviations in the manuscript have been fully written as requested in the revised manuscript.

18. When describing the maximum and minimum values of the attributes, the authors should use scored data.

Response: Thank you for your comment. We have included minimum, mean, and maximum values in Table 1. The information in this table was drawn from quantitative data.

19. Every caption should be enhanced.

Response: Thank you for your observation. We have enhanced the captions in the Tables and figures within the revised manuscript as requested.

20. The Delta K figure of genetic structure should be spotted

Response

We appreciate your comment. We have incorporated the information of obtained in the manuscript and added the optimal cluster information from the silhouette plot for our HC as a supplementary file.

21. The discussion did not have enough conviction. The analysis is a significant part of the remarks; yet, the majority of them merely repeat the findings without critically analyzing the facts. Please explain to readers how the authors' findings relate to those of other studies. Detailed information must be supplied on how each recorded parameter effects high or poor results. Their description should include all of the ramifications of setting high or low values for each of these parameters. The authors should interpret all measurements like PIC, He, and multivariate analysis.

Response: Thank you so much for your observation. We have revised the discussion section of the manuscript to ensure it provides adequate conviction to the readers as requested in the revised manuscript.

Conclusion

22. This section's authors should present a concise summary of the most important findings in an easy-to-read format. This area should be explored more in future studies

Response: Thank you for your observation. We have provided the summary of the most important findings in a concise manner as requested.
