# Supplementary material for: Genetic diversity and population structure of soybean (Glycine max (L.) Merril) germplasm
Source: PLoS One. 2025 May 8;20(5):e0312079. doi: 10.1371/journal.pone.0312079 (PMC12061401; doi:10.1371/journal.pone.0312079)
Supplement: Attachment — Submitted filename: Response to reviewers comments.docx [file pone.0312079.s005.docx]

**Genetic diversity and population structure of soybean (*Glycine max* (L.) Merril) germplasm**

Tenena Silue^1,2,^*, Paterne Angelot Agre^2^, Bunmi Olasanmi^3^, Adeyinka Saburi Adewumi^2^, Idris Ishola Adejumobi^2^ and Abush Tesfaye Abebe ^2,^*

**Authors’ Responses to Reviewers' comments**

Reviewer #1: Dear …
The researchers analyzed the genetic diversity in soybean accessions using DArt technology. There isn't much new information available on the subject. The following adjustments are needed:

**Abstract**

1. The aim and the methods should be joined into a single phrase

**Response**: Thanks for your valuable observation. The aim and the methods have been joined into a single phrase. (Reference: Lines 22-25).

2. It would be helpful to include a brief conclusion about the obtained results

**Response**: This has been adequately taken care off (Lines 33-35).

**Introduction**

1. This section is too long and should be summarized

**Response**: Thank you for your observation. This section has been reduced to provide the most relevant information.

2. It is suggested that you use the most current references that you can find.

**Response**: Thank you for this comment, we have included as many recent references that are relevant to our work as possible while retaining the old references that are highly important in the revised version of the manuscript.

3. The authors should describe the DNA molecular marker related to this study

**Response**: Thank you for your observation, the requested information has been provided. (Lines 84-97) in the revised manuscript.

4. Detailed information regarding methodologies for genetic diversity should be provided.

**Response**: Thank you for your observation. The methodologies for assessing genetic diversity in crops were documented in lines 68 – 83.

5. The authors should provide the gap and hypothesis of this study.

**Response**: Thank you for your comment. The requested information has been updated. (Lines 98-104).

6. The authors should expressly indicate in a single sentence the originality of the work

**Response**: Thank you for the comments. This aspect has been addressed. (Lines 104-106).

7. What distinguishes this study is that the authors identified new tasks or linkages related to their investigation.

**Response**: We appreciate your comments. We have included the statement showing how our work was unique following the identified gap in the IITA soybean breeding program.

8. Both general and specific goals should be documented

**Response**: Thank you for your comment. This observation has been provided (Lines 109-110).

**Material and methods**

9. The tissue type used for DNA extraction should be described

**Response**: Thank you for your comment. The description of the tissue type used for DNA extraction was documented in lines 123-128.

10. The name of the commercial kit should be included

**Response**: Thank you for your comment. We have included the names of the commercial kits as requested in the revised manuscript. (Lines 129-141).

11. It is essential to cite your sources or references for each approach

**Response**: Thank you for your observation. Appropriate citations following the format of the PLOSONE journal have been made for the sources and approaches we explored and documented in the revised version of the manuscript.

12. The manufacture of all instruments and materials should be stated

**Response**: Thank you for your observation. We have tried our best to include the manufacturer of the instruments and materials that we could find as requested in the revised version of the manuscript. Our limitation was that the genotyping procedure (DNA extraction and genomic scan) was all performed by the DArT laboratory.

13. Why did not the authors perform all measurements of genetic diversity indices like Na, Ne, and I?

**Response**: we appreciate your contribution in ensuring our manuscript is better improved. Our understanding of Na, Ne, and I points to population size, effective sample size, and inbreeding depression, respectively which we do not believe may provide newer and different information than the diversity parameters (allele frequency, PIC, H_O,_ H_e,_ 1/D, H’, and A) we have explored in the study.

14. The authors should perform the computation of the genetic diversity index for population

**Response**: Thank you so much for this suggestion. We have included more population diversity assessment indices including the Shannon-Wiever index, inverse Simpson index, and Alpha diversity index to verify the results obtained from the marker diversity indices.

**Results and discussion**

15. This section is simply written and should be detailed

**Response**: Thanks so much for your observation. We have included more interpretations and tailored our discussion to the interpreted results in the revised version of the manuscript.

16. The results of the tables should be detailed

**Response**: Thanks so much for your suggestion. The results in the tables have been improved to include a better caption and footnote adjustment in the revised version of the manuscript as requested.

17. All abbreviations should be written in full name

**Response**: Thank you for your comment. Abbreviations in the manuscript have been fully written as requested in the revised manuscript.

18. When describing the maximum and minimum values of the attributes, the authors should use scored data.

**Response**: Thank you for your comment. We have included minimum, mean, and maximum values in Table 1. The information in this table was drawn from quantitative data.

19. Every caption should be enhanced.

**Response**: Thank you for your observation. We have enhanced the captions in the Tables and figures within the revised manuscript as requested.

20. The Delta K figure of genetic structure should be spotted

**Response**

We appreciate your comment. We have incorporated the information of obtained in the manuscript and added the optimal cluster information from the silhouette plot for our HC as a supplementary file.

21. The discussion did not have enough conviction. The analysis is a significant part of the remarks; yet, the majority of them merely repeat the findings without critically analyzing the facts. Please explain to readers how the authors' findings relate to those of other studies. Detailed information must be supplied on how each recorded parameter effects high or poor results. Their description should include all of the ramifications of setting high or low values for each of these parameters. The authors should interpret all measurements like PIC, He, and multivariate analysis.

**Response**: Thank you so much for your observation. We have revised the discussion section of the manuscript to ensure it provides adequate conviction to the readers as requested in the revised manuscript.

**Conclusion**

22. This section's authors should present a concise summary of the most important findings in an easy-to-read format. This area should be explored more in future studies

**Response**: Thank you for your observation. We have provided the summary of the most important findings in a concise manner as requested.
