# Supplementary material for: Genetic diversity and population structure of soybean (Glycine max (L.) Merril) germplasm
Source: PLoS One. 2025 May 8;20(5):e0312079. doi: 10.1371/journal.pone.0312079 (PMC12061401; doi:10.1371/journal.pone.0312079)
Supplement: Attachment — Submitted filename: Comments.docx [file pone.0312079.s006.docx]

**Comments**

Authors of the study Genetic Diversity and Population Structure of Soybean germplasm has exhausted the manuscript well.

However they must take note of the following;

1. L42-43 needs reference.
2. L 64-66 can be removed since the study is not about identifying genes.
3. L 117-118 seems the wording is not making the figures to add up well. Kindly rephase.
